# Japanese Society for Cancer of the Colon and Rectum (JSCCR) guidelines 2016 for the treatment of colorectal cancer

**DOI:** 10.1007/s10147-017-1101-6

**Published:** 2017-03-27

**Authors:** Toshiaki Watanabe, Kei Muro, Yoichi Ajioka, Yojiro Hashiguchi, Yoshinori Ito, Yutaka Saito, Tetsuya Hamaguchi, Hideyuki Ishida, Megumi Ishiguro, Soichiro Ishihara, Yukihide Kanemitsu, Hiroshi Kawano, Yusuke Kinugasa, Norihiro Kokudo, Keiko Murofushi, Takako Nakajima, Shiro Oka, Yoshiharu Sakai, Akihito Tsuji, Keisuke Uehara, Hideki Ueno, Kentaro Yamazaki, Masahiro Yoshida, Takayuki Yoshino, Narikazu Boku, Takahiro Fujimori, Michio Itabashi, Nobuo Koinuma, Takayuki Morita, Genichi Nishimura, Yuh Sakata, Yasuhiro Shimada, Keiichi Takahashi, Shinji Tanaka, Osamu Tsuruta, Toshiharu Yamaguchi, Naohiko Yamaguchi, Toshiaki Tanaka, Kenjiro Kotake, Kenichi Sugihara, Japanese Society for Cancer of the Colon and Rectum

**Affiliations:** 1 Department of Surgical Oncology, The Graduate School of Medicine, The University of Tokyo, 7-3-1 Hongo, Bunkyo-ku, Tokyo, 113-8655 Japan; 2 Department of Clinical Oncology, Aichi Cancer Center Hospital, Nagoya, Japan; 3 Division of Molecular and Diagnostic Pathology, Graduate School of Medical and Dental Sciences, Niigata University, Niigata, Japan; 4 Department of Surgery, Teikyo University, Tokyo, Japan; 5 Department of Radiation Oncology, National Cancer Center Hospital, Tokyo, Japan; 6 Endoscopy Division, National Cancer Center Hospital, Tokyo, Japan; 7 Division of Gastrointestinal Medical Oncology, National Cancer Center Hospital, Tokyo, Japan; 8 Department of Digestive Tract and General Surgery, Saitama Medical Center, Saitama Medical University, Saitama, Japan; 9 Department of Translational Oncology, Tokyo Medical and Dental University Graduate School, Tokyo, Japan; 10 Colorectal Surgery Division, National Cancer Center Hospital, Tokyo, Japan; 11 Department of Gastroenterology, St. Mary’s Hospital, Fukuoka, Japan; 12 Department of Colon and Rectal Surgery, Shizuoka Cancer Center, Shizuoka, Japan; 13 Hepato-Biliary-Pancreatic Surgery Division, Artificial Organ and Transplantation Division, Department of Surgery, Graduate School of Medicine, University of Tokyo, Tokyo, Japan; 14 Radiation Oncology Department, The Cancer Institute Hospital, Japanese Foundation for Cancer Research, Tokyo, Japan; 15 Department of Clinical Oncology, St. Marianna University School of Medicine, Kawasaki, Japan; 16 Gastroenterology and Metabolism, Hiroshima University Hospital, Hiroshima, Japan; 17 Department of Surgery, Kyoto University, Kyoto, Japan; 18 Department of Clinical Oncology, Faculty of Medicine, Kagawa University, Takamatsu, Japan; 19 Division of Surgical Oncology, Department of Surgery, Nagoya University Graduate School of Medicine, Nagoya, Japan; 20 Department of Surgery, National Defense Medical College, Saitama, Japan; 21 Division of Gastrointestinal Oncology, Shizuoka Cancer Center, Shizuoka, Japan; 22 Department of Hemodialysis and Surgery, Chemotherapy Research Institute, International University of Health and Welfare, Ichikawa, Japan; 23 Department of Gastroenterology and Gastrointestinal Oncology, National Cancer Center Hospital East, Chiba, Japan; 24 Diagnostic Pathology Center, Shinko Hospital, Kobe, Japan; 25 Department of Surgery, Institute of Gastroenterology, Tokyo Women’s Medical University, Tokyo, Japan; 26 Department of Health Administration and Policy, Tohoku Medical and Pharmaceutical University, Sendai, Japan; 27 Department of Surgery, Cancer Center, Aomori Prefectural Central Hospital, Aomori, Japan; 28 Department of Surgery, Japanese Red Cross Kanazawa Hospital, Ishikawa, Japan; 29 CEO, Misawa City Hospital, Misawa, Japan; 30 Division of Clinical Oncolgy, Kochi Health Sciences Center, Kochi, Japan; 31 Department of Surgery, Tokyo Metropolitan Cancer and Infectious Diseases Center Komagome Hospital, Tokyo, Japan; 32 Department of Endoscopy, Hiroshima University Hospital, Hiroshima, Japan; 33 Division of GI Endoscopy, Kurume University School of Medicine, Fukuoka, Japan; 34 Department of Gastroenterological Surgery, The Cancer Institute Hospital, Japanese Foundation for Cancer Research, Tokyo, Japan; 35 Library of SEIREI SAKURA Citizen Hospital, Sakura, Japan; 36 Department of Surgery, Tochigi Cancer Center, Utsunomiya, Japan; 37 Koujinkai Daiichi Hospital, Tokyo, Japan

**Keywords:** Colorectal cancer, Guideline, Surgery, Chemotherapy, Endoscopy, Radiotherapy

## Abstract

Japanese mortality due to colorectal cancer is on the rise, surpassing 49,000 in 2015. Many new treatment methods have been developed during recent decades. The Japanese Society for Cancer of the Colon and Rectum Guidelines 2016 for the treatment of colorectal cancer (JSCCR Guidelines 2016) were prepared to show standard treatment strategies for colorectal cancer, to eliminate disparities among institutions in terms of treatment, to eliminate unnecessary treatment and insufficient treatment, and to deepen mutual understanding between health-care professionals and patients by making these Guidelines available to the general public. These Guidelines were prepared by consensus reached by the JSCCR Guideline Committee, based on a careful review of the evidence retrieved by literature searches, and in view of the medical health insurance system and actual clinical practice settings in Japan. Therefore, these Guidelines can be used as a tool for treating colorectal cancer in actual clinical practice settings. More specifically, they can be used as a guide to obtaining informed consent from patients and choosing the method of treatment for each patient. As a result of the discussions held by the Guideline Committee, controversial issues were selected as Clinical Questions, and recommendations were made. Each recommendation is accompanied by a classification of the evidence and a classification of recommendation categories based on the consensus reached by the Guideline Committee members. Here we present the English version of the JSCCR Guidelines 2016.

Introduction

1. Guideline objectives

Incidence and mortality of colorectal cancer have substantially increased in Japan recently. According to the vital statistics of Japan in 2015, colorectal cancer accounted for the largest number of deaths from malignant neoplasms in women. Nevertheless, the number of deaths from colorectal cancer per unit population has increased approximately tenfold during the past 50 years. Many new treatment methods have been developed during that time, and their use in combination with advances in diagnostic methods has led to a steady improvement in the results of treatment. However, there are differences in treatment among medical institutions in Japan that provide medical care for patients with colorectal cancer, and the differences may lead to differences in the results of treatment.

Under such circumstances, the JSCCR guidelines 2016 for the treatment of colorectal cancer (JSCCR Guidelines 2016), which are intended for doctors (general practitioners and specialists) who provide medical care for patients with colorectal cancer in various disease stages and conditions, were prepared for the following purposes: (1) To show standard treatment strategies for colorectal cancer, (2) To eliminate disparities among institutions in terms of treatment, (3) To eliminate unnecessary treatment and insufficient treatment, (4) To deepen mutual understanding between health-care professionals and patients by making these Guidelines available to the general public [1].

The following are expected to be achieved with these Guidelines: (1) Improvement of the treatment of colorectal cancer in Japan; (2) Improvement of the results of treatment; (3) Reduction of the human and financial burden; (4) Increased benefits for patients.

2. How to use these guidelines

These Guidelines were prepared by consensus reached by the Guideline Committee of the Japanese Society for Cancer of the Colon and Rectum, based on a careful review of the evidence retrieved by literature searches and in view of the medical health insurance system and actual clinical practice settings in Japan, and therefore, they can be used as a tool for treating colorectal cancer in actual clinical practice settings. More specifically, they can be used as a guide to obtaining informed consent from patients and choosing the method of treatment for each patient. However, these Guidelines provide only general recommendations for choosing treatment strategies for colorectal cancer, and they do not control or limit treatment strategies or treatment methods that are not described herein. They can also be used as a document to explain the rationale for selecting treatment strategies and treatment methods that differ from those described therein.

The Japanese Society for Cancer of the Colon and Rectum (JSCCR) is responsible for the statements in these Guidelines. However, the personnel directly in charge of treatment, not the JSCCR or the Guideline Committee, are responsible for the outcome of treatment.

3. Users

The users of these Guidelines are mainly clinical doctors engaged in all aspects of the medical treatment of colorectal cancer.

4. How to develop these Guidelines

(1) Recording methods

We adopted the concept from the first edition, in which the treatment policy algorithm was disclosed and a simple explanation thereof recorded and added further comments in regard to categories requiring additional explanation. Since the 2009 edition, areas of debate have been raised as clinical questions (CQs) and included with recommendations added. In the 2016 edition, this practice was continued, with corrections and additions made to the CQs based on knowledge acquired since the 2010 version.

(2) Evidence level/strength of recommendations of CQs

The recommendations added to CQs included the evidence level and strength of recommendations determined using the following direction.

(2-1) Evidence level

Papers relating to the CQs were comprehensively collected, and the evidence indicated by individual papers relating to the critical outcomes included within the CQs was divided into groups by study design [2]. The literature level and a body of evidence (Table 1) were evaluated in reference to the GRADE* System [3–25], before determining the final CQ evidence level (Table 2).

**Table 1:** Rating the quality of evidence

Step 1 (evaluation of individual study): study design, evaluation of bias risk, create structured abstract
Step 2 (overall rating for each important outcome across studies)
1. Initial quality of a body of evidence: evaluation of each study design group
systematic reviews, meta-analysis, randomized controlled trials = “initial quality A (high level)”
observation studies, cohort studies, case control studies = “initial quality C (low level)”
case series, case reports = “initial quality D (very low level)”
2. Five reasons to possibility rate down the quality
risk of bias
inconsistency in results
indirectness of evidence
data imprecision
high possibility of publication bias
3. Three reasons to possibility rate up the quality
large effect with no confounding factors
dose–response gradient
possible confounding factors are weaker than actual effects
4. We evaluate 1 → 2 → 3, and assess the quality of a body of evidence

**Table 2:** Definition of levels of evidence (Reference [13])

A (high)	We are very confident in the effect estimate
B (moderate)	We are moderately confident in the effect estimate: the true effect is likely to be close to the estimate of the effect, but there is a possibility that it is substantially different
C (low)	Our confidence in the effect estimate is limited: the true effect may be substantially different from the estimate of the effect
D (very low)	We have very little confidence in the effect estimate: the true effect is likely to be substantially different from the estimate of effect

*GRADE: The Grading of Recommendations Assessment, Development, and Evaluation.

(2-2) Strength of recommendations

Draft recommendation statements and the strength of the recommendations were directed based on the outcomes and the level of evidence obtained from the process described above and were evaluated at a consensus meeting of the Guideline Committee.

The draft recommendations were evaluated from four categories (① Quality of evidence, ② Patients’ views and preferences, ③ Benefits and harms, and ④ Cost effectiveness). The strength of recommendation (Table 3) was determined by vote, based on the Delphi method, with those reaching a consensus of opinion of 70% or more committee members determined as having been agreed upon. Items not reaching consensus after a single vote were debated once again, with the results of the first vote disclosed and additional information on the situation relating to clinical practice in Japan provided, and discussion and voting was repeated until a consensus was reached. No strength of recommendation was presented in CQs.

**Table 3:** Strength of recommendation (Reference [24])

Strength of recommendation	
1 (Strong recommendation)	Strong “For” an intervention
	Strong “Against” an intervention
2 (Weak recommendation)	Weak “For” an intervention
	Weak “Against” an intervention

5. Literature search

At first, the literature search was performed for the following 12 broad categories. Then a further search was done as needed with additional search techniques.

1. Endoscopic treatment of colorectal cancer
2. Treatment of Stage 0 to Stage III colorectal cancer [26]
3. Treatment of Stage IV colorectal cancer [26]
4. Treatment of liver metastases of colorectal cancer
5. Treatment of lung metastases of colorectal cancer
6. Treatment of recurrent colorectal cancer
7. Adjuvant chemotherapy for colorectal cancer
8. Chemotherapy for unresectable colorectal cancer
9. Adjuvant radiotherapy for colorectal cancer
10. Palliative radiotherapy for colorectal cancer
11. Palliative care for colorectal cancer
12. Surveillance after surgery for colorectal cancer

In order to survey the latest literature, in addition to the papers used for reference in the previous edition, the PubMed and Ichushi-Web databases were selected for the search, and the English and Japanese literature was searched in both databases from January 2008 to March 2012. However, the end of the search period for (7) and (8) was July 2016. The task of searching was shared by four members of the medical library; the four members created a search formula by discussion with the committee members in charge of each item and collected literature during the search period. In addition, secondary documents such as UpToDate and literature collected by manual searching were added and critically examined as needed, and other documents such as minutes and guidelines were included as necessary. We selected 2320 documents from among the 12,000 documents (PubMed 7909, ICHUSHI 4091) collected during the literature search and critically reviewed all of them (Table 4).

**Table 4:** Number of scientific articles retrieved and selected

	Number of articles retrieved		Number of articles selected		Number of articles retrieved manually
	PubMed	Ichushi	PubMed	Ichushi	
(1) Endoscopic treatment of colorectal cancer	811	385	80	40	39
(2) Treatment of Stage 0 to Stage III colorectal cancer	469	285	92	14	12
(3) Treatment of Stage IV colorectal cancer	237	102	97	14	13
(4) Treatment of liver metastases of colorectal cancer	812	357	364	79	25
(5) Treatment of lung metastases of colorectal cancer	96	157	46	35	6
(6) Treatment of recurrent colorectal cancer	688	302	147	29	13
(7) Adjuvant chemotherapy for colorectal cancer	855	450	244	39	47
(8) Chemotherapy for advanced or recurrent colorectal cancer	1062	451	320	53	157
(9) Adjuvant radiotherapy for colorectal cancer	447	95	115	8	27
(10) Palliative radiotherapy for colorectal cancer	708	39	109	6	29
(11) Palliative care for colorectal cancer	278	181	58	18	10
(12) Surveillance after surgery for colorectal cancer	1446	1287	256	57	20
Total	7909	4091	1928	392	398

Treatment guidelines for colorectal cancer

Chapter 1: Treatment strategies for Stage 0 to Stage III colorectal cancer [26]

1. Endoscopic treatment (Fig. 1)

General principles underlying the indications for endoscopic resection.

**Fig. 1:**
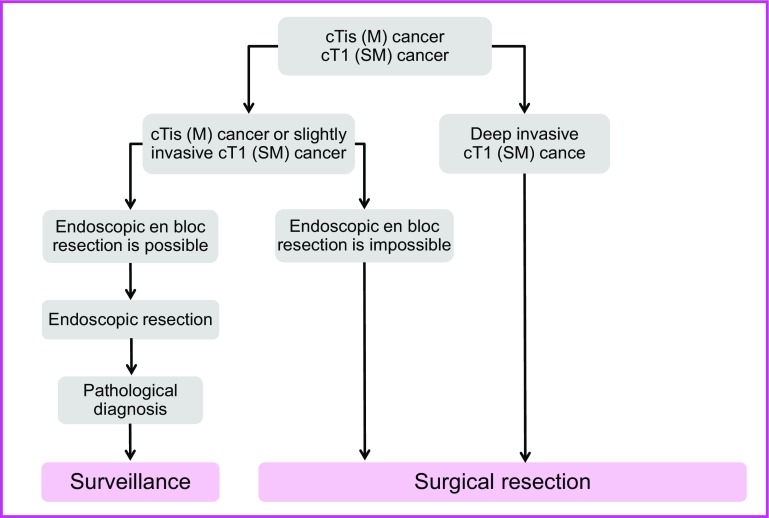
Treatment strategies for cTis (M) cancer and cT1 (SM) cancer

- There is little possibility of lymph node metastasis, and the size and location of the tumor make en bloc resection possible.

Indication criteria for endoscopic resection:

1. Intramucosal carcinoma or carcinoma with slight submucosal invasion
2. Size does not matter
3. Any macroscopic type
  Endoscopic treatment is a method of endoscopically resecting lesions in the large bowel and of collecting the resected specimens.
  Endoscopic treatment methods consist of polypectomy (note 1), endoscopic mucosal resection (EMR) (note 2), and endoscopic submucosal dissection (ESD) (note 3).
  In determining the indication for endoscopic treatment and the treatment method, information on the size, predicted depth of invasion, and morphology of the tumor is essential.

Comments

① Endoscopic resection is intended for both diagnosis and treatment. It consists of total excisional biopsy in which curability and the necessity of additional intestinal resection are assessed by histopathological examination of the resected specimens. (CQ-1)
② En bloc resection is desirable for accurate diagnosis of the status of carcinoma invasion in the resection margin and the deepest area.
  2 cm is the largest size of a tumor that can be easily resected en bloc by polypectomy or snare EMR [27]. (CQ-2)
  Colorectal ESD is an “endoscopic resection technique which enables en bloc resection of a tumor, regardless of size,” which was approved for implementation under health insurance in April 2014 in regard to “early-stage malignant tumors” Given the high likelihood of technically difficult complications (perforations); however, it should only be implemented after sufficient consideration of the level of skill of the endoscopist performing the procedure. At present, tumors with a diameter between 2-5 cm are covered by insurance (CQ-3).
  EMRC (EMR using a cap) is reported to involve a high risk of perforation when used for colon lesions.
  If the preoperative diagnosis is cancer accompanied by adenoma (intramucosal carcinoma), a piecemeal resection can be performed in regard to the adenoma, while avoiding division of the cancerous area. It should be noted, however, that piecemeal resection is associated with a high incomplete resection rate and a high local recurrence rate [27].

Note 1: Polypectomy—In this method, a snare is placed on the stalk of the lesion, and the lesion is electrocauterized using a high-frequency current. This method is mainly used for protruding lesions
Note 2: EMR—In this method, the lesion is elevated by local injection of a liquid such as physiological saline into the submucosa, and the lesion is electrocauterized the same as in case of polypectomy. This method includes the snare method [3] and EMR using a cap (EMRC). It is mainly used for superficial tumors and large sessile lesions
Note 3: ESD—In this technique, the lesion is elevated by local injection of a liquid such as sodium hyaluronate solution into the submucosa of the perilesional area; then, circumferential incision of the mucosa surrounding the lesion and dissection of the submucosa with a special knife and en bloc resection are performed [28]. ESD is mainly indicated for large tumors, especially for early cancers that cannot be resected by EMR.

2. Surgical treatment (Fig. 2)

- The extent of lymph node dissection to be performed during colorectal cancer surgery is determined based on the preoperative clinical findings and on the extent of lymph node metastasis and depth of tumor invasion by the tumor observed intraoperatively.
- If lymph node metastasis is recognized, or suspected based on the preoperative/intraoperative findings, D3 dissection is performed.
- If no lymph node metastases are observed based on the preoperative/intraoperative diagnostic findings, lymph node dissection is performed based on the depth of tumor invasion [29].

**Fig. 2:**
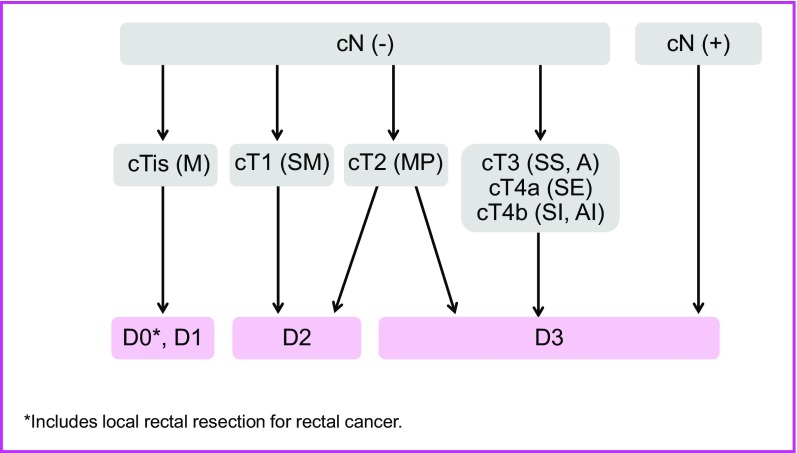
Surgical treatment strategies for cStage 0 to cStage III colorectal cancer

1. Lymph node dissection is unnecessary for pTis (M) cancer (D0), because pTis (M) cancer is not accompanied by lymph node metastasis; however, D1 dissection can be performed because the accuracy of the preoperative diagnosis of invasion depth may be insufficient.
2. D2 dissection is necessary for pT1 (SM) cancer, because the incidence of lymph node metastasis is approximately 10% and because pT1 (SM) cancer is often accompanied by intermediate lymph node metastasis.
3. Although there is insufficient evidence describing the extent of lymph node dissection for cT2 (MP) cancer, at least D2 dissection is necessary. However, D3 dissection can be performed, because about 1% of cT2 (MP) cancer is accompanied by main lymph node metastases (Table 5) and because preoperative diagnosis of depth of invasion is not very accurate.

**Table 5:** Lateral dissection and lateral metastasis of rectal cancer

	No. of patients	No. of patients who underwent lateral dissection	Lateral dissection rate	No. of patients with lateral metastasis	Lateral metastasis rate (percentage of all patients)	Lateral metastasis rate (percentage of patients who underwent lateral dissection)
RS						
sm	124	0	0	0	0.0	0.0
mp	127	6	4.7%	0	0.0	0.0
ss/a_1_	316	24	7.5%	0	0.0	0.0
se/a_2_	177	8	4.5%	0	0.0	0.0
si/ai	32	14	43.8%	1	3.1	7.1
Total	776	52	6.7%	1	0.1	1.9
Ra						
sm	138	5	3.6%	0	0.0	0.0
mp	149	18	12.1%	0	0.0	0.0
ss/a_1_	230	58	25.2%	4	1.7	6.9
se/a_2_	181	59	32.6%	7	3.9	11.9
si/ai	15	8	53.3%	0	0.0	0.0
Total	713	148	20.8%	11	1.5	7.4
RaRb + Rb						
sm	234	37	15.8%	2	0.9	5.4
mp	372	218	58.6%	20	5.4	9.2
ss/a_1_	350	230	65.7%	28	7.7	12.2
se/a_2_	412	319	77.4%	75	18.0	23.5
si/ai	59	48	81.4%	17	28.8	35.4
Total	1427	852	59.7%	142	9.8	16.7

Project study by the JSCCR: patients in years 1991–1998

Surgical treatment for rectal cancer:

- The principle for radical surgery for rectal cancer is TME (total mesorectal excision) or TSME (tumor-specific mesorectal excision) [30–33].

[Indications criteria for lateral lymph node dissection]

- Lateral lymph node dissection is indicated when the lower border of the tumor is located distal to the peritoneal reflection and the tumor has invaded beyond the muscularis propria [30].

[Local excision for rectal cancer]

- Local excision is indicated for cTis (M) cancer and cT1 (SM) cancer (slight invasion) located distal to the second Houston valve (peritoneal reflection).
- Histological investigation of the resected specimen allows a determination to be made of the likelihood that treatment will cure the condition completely, along with the need for additional treatment (intestinal resection accompanied by lymph node dissection).

[Autonomic nerve-preserving surgery]

- The autonomic nervous system related to surgery for rectal cancer consists of the lumbar splanchnic nerves, superior hypogastric plexus, hypogastric nerves, pelvic splanchnic nerves, and pelvic plexus. Considering factors such as the degree of cancer progression and presence or absence of macroscopic nerve invasion, preservation of autonomic nerves is attempted in order to preserve urinary and sexual functions as much as possible, provided that curability is unaffected.

Laparoscopic surgery:

- The indications for laparoscopic surgery are determined by considering the surgeon’s experience and skills, as well as tumor factors, such as the location and degree of progression of the cancer, and patient factors, such as obesity and history of open abdominal surgery. (CQ-4)

Comments

[Lateral lymph node dissection]

① An analysis of 2916 cases of rectal cancer in the project study by the JSCCR showed that the lateral lymph node metastasis rate in patients whose lower tumor border was located distal to the peritoneal reflection and whose cancer invaded beyond the muscularis propria was 20.1% (only patients who underwent lateral lymph node dissection) (Table 5). After performing lateral lymph node dissection for the above mentioned indication, it is expected that the risk of intrapelvic recurrence decreases by 50%, and the 5-year survival rate improves by 8–9% [34].
② The lateral lymph node metastasis rate of patients whose lower tumor border was located distal to the peritoneal reflection and who had lymph node metastasis in the mesorectum was 27%.
③ Urinary function and male sexual function may be impaired after lateral dissection, even if the autonomic nervous system is completely preserved.

[Aggregate data from the colorectal cancer registry]

① The incidence of lymph node metastasis according to site and depth of tumor invasion, curative resection rate, and 5-year survival rate are shown in Tables 6, 7, and 8 [29].

**Table 6:** Incidences of lymph node metastasis according to primary site and depth of tumor invasion

	No. of patients	Extent of lymph node metastasis detected histologically				
		*n*_0_ (%)	*n*_1_ (%)	*n*_2_ (%)	*n*_3_ (%)	*n*_4_ (%)
All sites						
sm	3151	90.7	7.3	1.9	0.0	0.1
mp	3590	77.3	17.4	4.2	0.9	0.3
ss/a_1_	11,272	54.6	29.9	12.0	2.3	1.2
se/a_2_	6101	35.9	34.4	20.2	5.7	3.8
si/ai	1502	43.0	27.6	16.4	6.7	6.3
Total	25,617	57.1	26.3	11.9	2.9	1.9
Colon						
sm	1957	91.4	6.8	1.8	0.0	0.0
mp	1747	79.3	16.3	3.5	0.6	0.3
ss/a_1_	7333	56.6	28.1	11.7	2.4	1.2
se/a_2_	3363	37.4	34.0	19.3	5.6	3.7
si/ai	960	44.6	28.6	14.7	5.5	6.6
Total	15,360	58.6	25.4	11.3	2.8	1.8
Rectosigmoid						
sm	337	88.7	9.5	1.8	0.0	0.0
mp	429	80.4	17.0	2.6	0.0	0.0
ss/a_1_	1584	53.9	33.0	10.2	1.3	1.7
se/a_2_	789	34.2	38.4	20.8	3.2	3.4
si/ai	187	44.9	24.6	19.3	4.8	6.4
Total	3326	55.7	29.3	11.4	1.6	2.0
Rectum						
sm	839	89.7	7.7	2.0	0.1	0.4
mp	1373	73.9	19.2	5.4	1.4	0.1
ss/a_1_	2310	48.8	33.3	14.2	2.7	1.0
se/a_2_	1904	33.9	33.6	21.5	6.8	4.1
si/ai	328	38.1	26.2	19.8	10.4	5.5
Total	6754	54.3	27.0	13.3	3.6	1.8
Anal canal						
sm	18	94.4	0.0	5.6	0.0	0.0
mp	41	70.7	9.8	7.3	7.3	4.9
ss/a_1_	45	60.0	22.2	8.9	6.7	2.2
se/a_2_	46	32.6	21.7	23.9	15.2	6.5
si/ai	27	33.3	25.9	14.8	18.5	7.4
Total	177	54.8	17.5	13.0	10.2	4.5

National registry of patients with cancer of the colon and rectum of the JSCCR: patients in years 2000–2004. Depth of invasion and the degree of lymph node metastasis were determined according to the rules set forth in the “Japanese Classification of Colorectal Carcinoma” (6th edition)

**Table 7:** Curative resection rate according to stage (lower rows: no. of patients)

Stage	I	II	IIIa	IIIb	IV	All Stages
All patients	98.7%	96.2%	91.9%	81.8%	–	78.0%
	5455	7336	5635	2572	4300	25,298
Colon	99.1%	96.6%	92.4%	83.6%	–	77.2%
	3028	4688	3208	1379	2787	15,090
Rectosigmoid	99.5%	96.6%	92.5%	80.2%	–	78.0%
	615	961	835	288	560	3259
Rectum	97.9%	95.0%	90.9%	80.5%	–	79.9%
	1764	1644	1564	866	929	6767
Anal canal	95.8%	86.0%	78.6%	61.5%	–	70.9%
	48	43	28	39	24	182

National registry of patients with cancer of the colon and rectum of the JSCCR: patients in years 2000–2004

Curative resection rate = Number of patients with histological curability A cancer/Total number of patients who underwent surgery

Staging was performed according to the rules set forth in the “Japanese Classification of Colorectal Carcinoma” (6th edition)

**Table 8:** Cumulative 5-year survival rate according to site (lower rows: no. of patients)

Stage	0	I	II	IIIa	IIIb	IV	All Stages
Cecum	91.0%	93.7%	83.5%	73.0%	65.4%	12.5%	68.2%
	79	185	249	207	113	204	1037
Ascending colon	93.9%	91.2%	85.8%	79.1%	63.4%	19.1%	71.4%
	125	338	656	416	211	410	2156
Transverse colon	88.9%	91.4%	85.2%	78.5%	65.7%	20.8%	74.0%
	105	277	428	244	138	210	1402
Descending colon	100.0%	94.1%	85.3%	82.0%	52.9%	21.1%	75.4%
	43	146	224	166	52	117	748
Sigmoid colon	94.2%	92.3%	85.8%	83.0%	64.7%	22.0%	73.7%
	154	852	1124	837	363	736	4066
Rectosigmoid	89.4%	91.5%	84.8%	78.0%	60.0%	19.8%	71.6%
	54	366	539	473	175	322	1929
Upper rectum	98.0%	95.3%	84.6%	75.9%	57.7%	11.6%	72.4%
	67	356	464	471	173	263	1794
Lower rectum	97.5%	88.3%	81.7%	70.0%	51.4%	11.6%	70.5%
	142	718	486	473	332	298	2449
Anal canal	100.0%	78.7%	90.9%	46.9%	61.2%	15.7%	60.0%
	4	16	14	16	19	17	86
Colon	93.0%	92.3%	85.4%	80.4%	63.8%	19.9%	72.8%
	506	1798	2681	1870	877	1677	9409
Rectum	97.6%	90.6%	83.1%	73.0%	53.5%	14.8%	71.3%
	209	1074	950	944	505	561	4243
All sites	94.0%	91.6%	84.8%	77.7%	60.0%	18.8%	72.1%
	773	3254	4184	3303	1576	2577	15,667

National registry of patients with cancer of the colon and rectum of the JSCCR: patients in years 2000–2004

Only adenocarcinomas (including mucinous carcinomas and signet-ring cell carcinomas) were counted

Survival rates were calculated by the life table method with death from any cause as an event

5-year censoring rate = 20.5% (3208/15,667)

Staging was performed according to the rules set forth in the “Japanese Classification of Colorectal Carcinoma” (6th edition)
② The 5-year survival rates after curative resection of pStage 0 to pStage III colorectal cancer according to site were: All sites: 82.2%, Colon: 83.8%, Rectosigmoid: 81.7%, Ra-Rb rectum: 79.3% (patients in years 2000–2004).

Chapter 2: Treatment strategies for stage IV colorectal cancer [26] (Fig. 3)

- Stage IV colorectal cancer is associated with synchronous distant metastasis to any of the following organs: liver, lung, peritoneum, brain, distant lymph nodes, or other organ (e.g., bone, adrenal gland, spleen).
- If both the distant metastases and the primary tumor are resectable, curative resection of the primary tumor is performed, and resection of the distant metastases is considered.
- If the distant metastases are resectable, but the primary tumor is unresectable, in principle, resection of the primary tumor and distant metastases is not performed, and another treatment method is selected.
- If the distant metastases are unresectable, but the primary tumor is resectable, the indication for the resection of the primary tumor is determined, based on the clinical symptoms of the primary tumor and the impact on the prognosis (CQ-4).

**Fig. 3:**
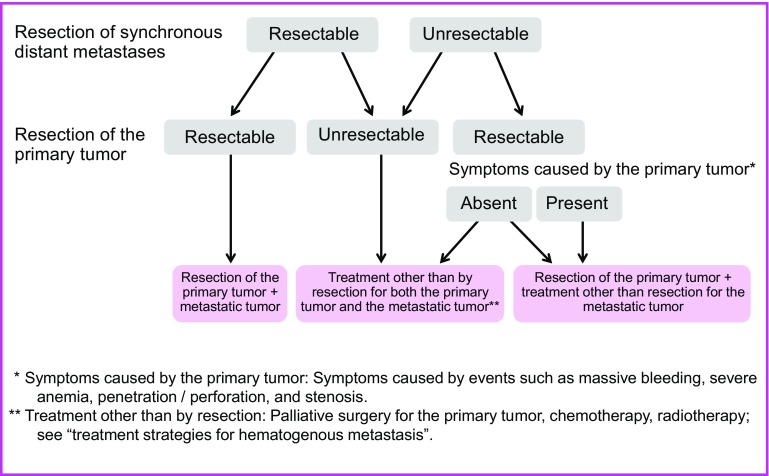
Treatment strategies for Stage IV colorectal cancer

Comments

① The incidence of synchronous distant metastasis is shown in Table 9.

**Table 9:** Incidence of synchronous distant metastasis of colorectal cancer

	Liver	Lung	Peritoneum	Other sites				
				Bone	Brain	Virchow	Other	Total
Colon cancer	11.8%	2.2%	5.7%	0.3%	0.0%	0.1%	1.3%	1.8%
No. of patients 15,391	1815	338	875	47	6	23	205	281
Rectal cancer	9.5%	2.7%	2.6%	0.5%	0.0%	0.1%	1.1%	1.7%
No. of patients 10,221	970	273	266	49	5	6	112	172
Total no. of patients	10.9%	2.4%	4.5%	0.4%	0.0%	0.1%	1.2%	1.8%
	2785	611	1141	96	11	29	317	453

National registry of patients with cancer of the colon and rectum of the JSCCR: patients in years 2000–2004
② Distant metastasis associated with peritoneal dissemination (CQ-6)
  Complete resection is desirable for P1.
  Complete resection is considered for P2 when easily resectable.
  The efficacy of resection of P3 has not been demonstrated.
③ Cases accompanied by distant metastasis to multiple organs
  Typically, these cases involve metastasis to the liver or lungs.
  If it is safe and simple to remove the primary lesion and the metastasized lesions in the liver or lungs, resection should also be considered [35, 36]. (CQ-7)
④ Adjuvant therapy subsequent to the resection of distant metastasis
  The efficacy and safety of adjuvant chemotherapy following the resection of distant metastasis in colorectal cancer have not been established, and no randomized controlled trials have been implemented regarding whether or not it extends survival [37, 38]. (CQ-8) Ideally, appropriately planned clinical trials should be implemented.

Chapter 3: Treatment strategies for recurrent colorectal cancer (Fig. 4)

- The goal of treatment for recurrent colorectal cancer is improvement of the prognosis and patient’s QOL.
- Treatment methods include surgery, systemic chemotherapy, arterial infusion chemotherapy, thermal coagulation therapy, and radiotherapy.
- An appropriate treatment method is selected with the informed consent of the patient in view of a variety of factors, such as the prognosis, complications, and QOL expected after treatment.
- If recurrence is observed in a single organ and complete surgical resection of the recurrent tumor(s) is possible, resection is strongly considered.
- If recurrence is observed in more than a single organ, resection can be considered if the recurrent tumors in all of the organs are resectable [35, 39]; however, there is no consensus on the effects of treatment (CQ-7).
- Some authors believe that resection of liver or lung metastases should be performed only after a certain observation period to rule out occult metastases [40].
- Systemic chemotherapy is effective in regard to cases of inoperable liver metastasis, with some cases demonstrating that curative resection may become possible [41, 42] (CQ-9).
- Treatment methods for hematogenous metastases (see chapter 4 “Treatment strategies for hematogenous metastases”)
- Local recurrences of rectal cancer take the form of anastomotic recurrences and intrapelvic recurrences.

**Fig. 4:**
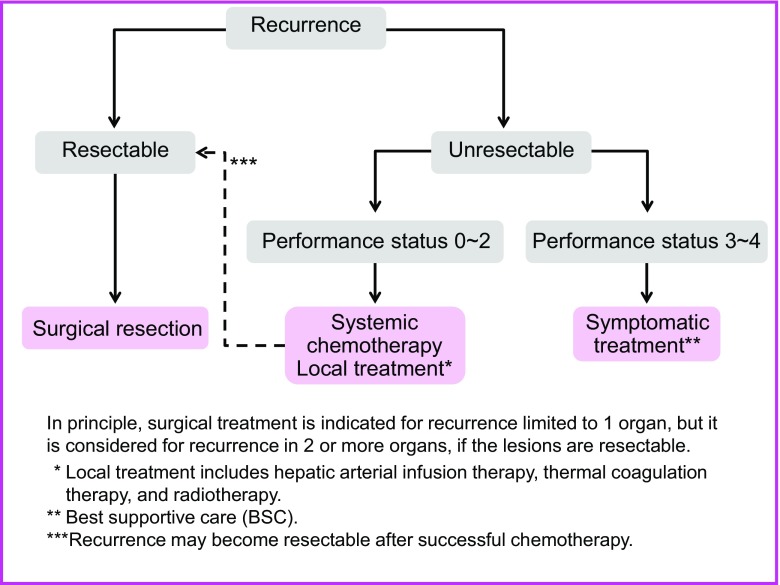
Treatment strategies for recurrent colorectal cancer

1. Resection is considered for resectable recurrences.
2. Radiotherapy and systemic chemotherapy, either alone or in combination, are considered for unresectable recurrences.

Comments

[Local recurrence of rectal cancer]

①. The extent of spread of the recurrent tumor is evaluated by diagnostic imaging, and resection is considered only for patients in whom complete resection can be expected, after taking into consideration such factors as the pattern of recurrence, symptoms, and physical findings (CQ-10).

Chapter 4: treatment strategies for hematogenous metastases (Fig. 5)

1. Treatment strategies for liver metastases

- Treatment of liver metastases is broadly divided into hepatectomy, systemic chemotherapy, hepatic arterial infusion therapy, and thermal coagulation therapy.
- Hepatectomy is recommended for liver metastases when curative resection is possible.
- Hepatectomy consists of systematic resection and partial (non-systematic) resection.
- Indication criteria for hepatectomy

**Fig. 5:**
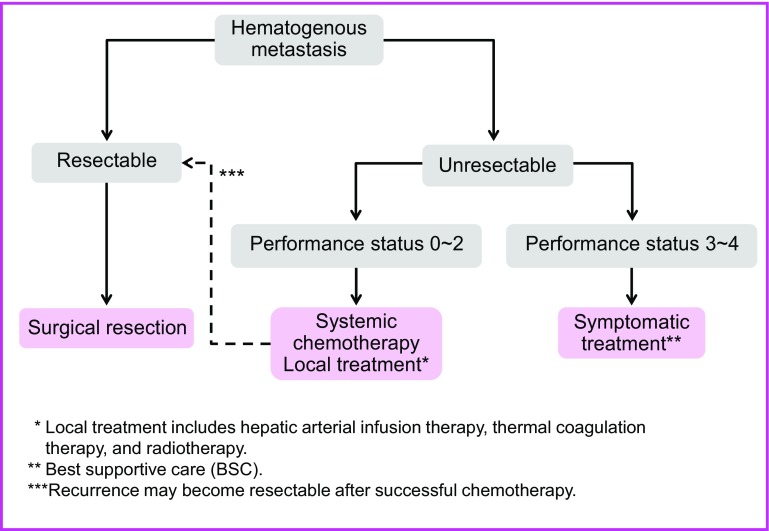
Treatment strategies for hematogenous metastases

1. The patient is capable of tolerating surgery
2. The primary tumor has been controlled or can be controlled.
3. The metastatic liver tumor can be completely resected.
4. There are no extrahepatic metastases or they can be controlled.
5. The function of the remaining liver will be adequate.
  Systemic chemotherapy is considered for patients with unresectable liver metastases whose general condition can be maintained at a certain level or higher (PS 0 to PS 2).
  Thermal coagulation therapy consists of microwave coagulation therapy (MCT) and radiofrequency ablation (RFA).
  If the patient’s general condition is poor (PS ≥3), or there is no effective chemotherapy, best supportive care (BSC) is provided.

Comments

[Hepatectomy]

① There are reports showing the efficacy of hepatectomy in patients who have controllable extrahepatic metastases (mainly lung metastases) in addition to liver metastases [35, 36, 39, 43] (CQ-7).
② The efficacy of systemic chemotherapy and hepatic arterial infusion therapy after hepatectomy has not been established (CQ-8).
③ The safety of preoperative chemotherapy for resectable liver metastases has not been established (CQ-11).

[Treatment methods other than resection]

① Systemic chemotherapy is performed for patients with unresectable liver metastases (CQ-9).
② In cases of inoperable liver metastasis, the primary lesion should ideally be managed if hepatic arterial infusion therapy or heat coagulation therapy is being used (CQ-17, CQ-12).
③ Heat coagulation therapy is advantageous in that it is minimally invasive, in addition to having been reported as improving local control and long-term survival in some cases [44, 45]. However, there have not yet been any studies or reports of long-term prognosis involving sufficiently cumulative case studies; consequently, its efficacy has not been established. There is a high rate of recurrence in comparison to resection, however, and long-term survival is reported to be poor [46], so it is not recommended as an alternative to surgical resection [47] (CQ-12).

2. Treatment strategies for lung metastases

- Treatment of lung metastases consists of pneumonectomy and systemic chemotherapy, and radiotherapy.
- Pneumonectomy is considered if the metastatic lung tumor is resectable.
- Pneumonectomy consists of systematic resection and partial (non-systematic) resection.

Indication criteria for pneumonectomy

1. The patient is capable of tolerating surgery.
2. The primary tumor has been controlled or can be controlled.
3. The metastatic lung tumor can be completely resected.
4. There are no extrapulmonary metastases or they can be controlled.
5. The function of the remaining lung will be adequate.
  Systemic chemotherapy is considered for patients with unresectable lung metastases whose general condition can be maintained at a certain level or higher.
  Even if the patient cannot tolerate surgery, stereotactic body radiotherapy is considered if the primary tumor and extrapulmonary metastases are controlled or can be controlled and the number of lung metastases within 5 cm in diameter is no more than three [48].
  If the patient’s general condition is poor, appropriate BSC is provided.

3. Treatment strategies for brain metastases

- Brain metastases are often detected as a part of a systemic disease, and surgical therapy or radiotherapy is considered for lesions in which treatment can be expected to be effective.
- The optimal treatment method is selected after considering the patient’s general condition and status of other metastatic tumors, and evaluating the size and location of metastatic brain tumors and the number of brain lesions.
- Radiotherapy is considered for patients with unresectable metastases.

[Surgical therapy]

Indications criteria for brain resection [49]

1. The patient has a life expectancy of at least several months.
2. Resection will not cause significant neurologic symptoms.
3. There are no metastases to other organs or they can be controlled.

[Radiotherapy]

- The purpose of radiotherapy is to relieve symptoms, such as cranial nerve symptoms and intracranial hypertension symptoms, and to prolong survival time by reducing locoregional relapse.
- Whole-brain radiotherapy is considered for patients with multiple brain metastases and for patients with a solitary brain metastasis for which surgical resection is not indicated.
- Stereotactic irradiation is considered when the number of brain metastases is about no more than three or four and the maximum diameter of each metastasis does not exceed 3 cm.

4. Treatment strategies for hematogenous metastases to other organs

- Resection is also considered for other hematogenous metastases, such as to the adrenal glands, skin, and spleen, if they are resectable. However, patients with such metastases often have metastasis to more than one organ, and chemotherapy or radiotherapy is often indicated.

Chapter 5: Chemotherapy

- Chemotherapy consists of adjuvant chemotherapy to prevent postoperative recurrence and systemic chemotherapy to treat unresectable colorectal cancer.
- Commonly used anticancer drugs that have been approved for the indication of colorectal cancer and are covered by the Japanese National Health Insurance include the following:

Oral drugs: 5-FU, tegafur, UFT, doxifluridine (5′-DFUR), carmofur (HCFU), S-1 (S), UFT + leucovorin (LV), capecitabine (Cape), regorafenib, trifluridine–tipiracil hydrochloride (TAS-102), etc.
Injectable drugs: 5-FU, mitomycin C, irinotecan (IRI), 5-FU + *l*-leucovorin (*l*-LV), oxaliplatin (OX), bevacizumab (Bmab), ramucirumab (Rmab), cetuximab (Cmab), panitumumab (Pmab), etc.

1. Adjuvant chemotherapy

- Postoperative adjuvant chemotherapy is a systemic chemotherapy that is performed after surgery to prevent recurrence and improve the prognosis of patients who have undergone R0 resection [50].

General principles for the indications of adjuvant chemotherapy

1. Stage III colorectal cancer (colon and rectal cancer) for which R0 resection has been performed. See CQ-8 for Stage IV resection cases.
2. The function of major organs is maintained as provided by the following guidelines:
  Bone marrow: Peripheral blood neutrophil count >1500/mm^3^; platelet count >100,000/mm^3^
  Liver function: Total bilirubin <2.0 mg/dL; AST/ALT <100 IU/L
  Renal function: Serum creatinine concentration is no higher than the upper limit of the normal range at the institution.
3. Performance status (PS) of 0 or 1.
4. The patient has recovered from postoperative complications, if any.
5. The patient has provided written informed consent.
6. The patient has no serious complications (particularly intestinal obstruction, diarrhoea, or fever).
  For age, see CQ-13.
  For patients who have Stage II colorectal cancer with a high risk of recurrence, the indications for adjuvant chemotherapy are considered after obtaining informed consent [51, 52] (CQ-14).

Recommended therapies (listed in the order of their date of coverage by the Japanese National Health Insurance)

- 5-FU + *l*-LV (note)
- UFT + LV
- Cape
- FOLFOX
- CapeOX
- S-1

Recommended administration period (CQ-15)

- In principle, the administration period is 6 months.

Note: The Roswell Park Memorial Institute (RPMI) method of 5-FU + LV therapy as adjuvant chemotherapy (drip infusion of *l*-LV 250 mg/m^2^ administered for 2 h; intravenous infusion of 5-FU 500 mg/m^2^ slowly administered within 3 min at 1 h after initiating *l*-LV administration; and once weekly administration for six consecutive weeks followed by a 2-week rest period, three cycles every 8 weeks [53]).

2. Chemotherapy for unresectable colorectal cancer (Fig. 6)

- In the best supportive care (BSC) without any chemotherapy, the median survival time (MST) of patients with unresectable colorectal cancer has been reported to be approximately 8 months. Although their MST has been extended to approximately 30 months because of recent chemotherapy, unresectable colorectal cancer remains difficult to cure.
- The purpose of chemotherapy is to prolong survival time and control symptoms by delaying tumour enlargement.
- Randomized controlled trials involving PS 0 to PS 2 patients have shown that chemotherapy groups have a significantly longer survival time than BSC groups that did not receive anticancer drugs [54–56].
- Initially unresectable colorectal cancer may become resectable after successful chemotherapy.
- Patients should be ideally divided into two groups and their treatment policy selected according to whether or not they are appropriate for intensive therapy.
- Patients appropriate for intensive therapy include those with no serious comorbidities who are considered tolerant to primary treatment with OX and IRI, as well as concomitant therapy with molecular target drugs. These patients, who have considerably slow tumour advancement and have preferably not suffered severe adverse events can be treated with either monotherapy or doublet therapy as the primary treatment.
- Patients inappropriate for intensive therapy include those with serious comorbidities who are considered intolerant to primary treatment with OX and IRI, as well as concomitant therapy with molecular target drugs. For these patients, monotherapy or doublet therapy shall be considered as the primary treatment.
- Cmab and Pmab are only used in response to wild-type RAS(KRAS/NRAS).
- Combination with molecular target drugs, such as Bmab, anti-EGFR antibodies, or Rmab, is recommended, but for patients who are not candidates, chemotherapy alone is administered.

**Fig. 6:**
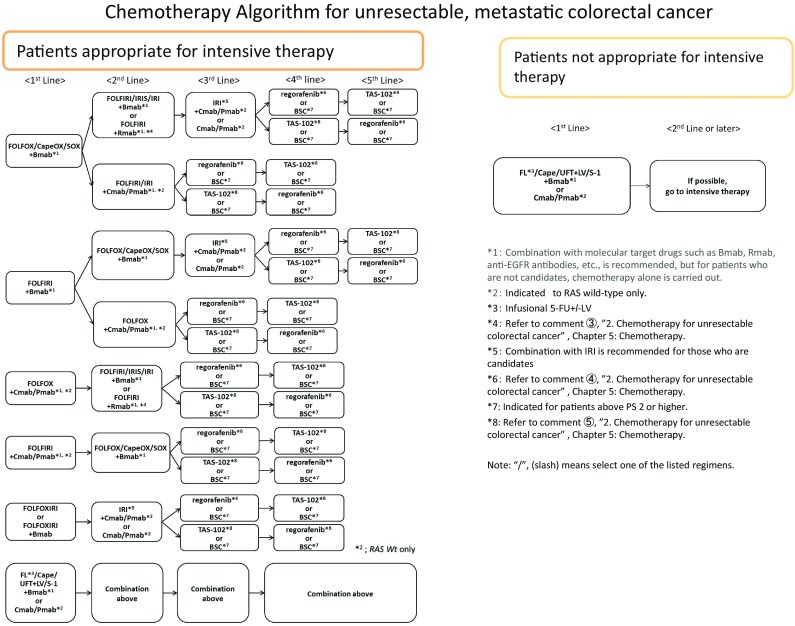
Chemotherapy for unresectable colorectal cancer

General principles underlying the indications of systemic chemotherapy

1. The clinical or histopathological diagnosis has been confirmed.
2. The metastatic or recurrent tumour can be confirmed by imaging.
3. Performance status (PS) is 0–2.
4. The function of major organs is maintained. (See 1–3 below for administration guidelines).
5. Bone marrow: Peripheral blood neutrophil count ≧1500/mm^3^; platelet count ≧100,000/mm^3^
6. Liver function: Total bilirubin <2.0 mg/dL; AST/ALT <100 IU/L
7. Renal function: Serum creatinine concentration is no higher than the upper limit of the normal range at the institution
8. The patient has provided written informed consent.
9. The patient has no serious complications (particularly intestinal obstruction, diarrhoea, or fever).

First-line therapy

- The following are regimens whose usefulness has been demonstrated in clinical trials. They are also available as the initial therapy covered by the Japanese National Health Insurance.

1. Patients appropriate for intensive therapy
  FOLFOX (note 1) [57, 58] + Bmab [54]
  CapeOX (note 2) + Bmab [59, 60]
  SOX + Bmab (note 3) [61]
  FOLFIRI (note 4) [62, 63] + Bmab [64, 65]
  FOLFOX + Cmab/Pmab [66, 67]
  FOLFIRI + Cmab/Pmab [68, 69]
  FOLFOXIRI (note 5) [70]
  FOLFOXIRI + Bmab [71, 72]
  Infusional 5-FU + LV [73, 74] + Bmab [75, 76]
  Cape [77, 78] + Bmab [79]
  UFT + LV [80–82] + Bmab
  S-1 + Bmab
  Cmab/Pmab [83, 84]
2. Patients not appropriate for intensive therapy
  Infusional 5-FU + LV + Bmab [75, 76]
  Cape + Bmab
  UFT + LV + Bmab
  S-1 + Bmab
  Cmab/Pmab

Second-line therapy

- The following regimens are considered as chemotherapy for second-line therapy (CQ-16).

- Patients appropriate for intensive therapy
- When the patient has become refractory or intolerant to the first-line therapy, including OX
  FOLFIRI [62] + Bmab [85]
  FOLFIRI + Rmab [86]
  IRIS (note 6) [87] + Bmab
  IRI [88] + Bmab
  FOLFIRI (or IRI) + Cmab/Pmab [88, 89]

(b) When the patient has become refractory or intolerant to the first-line therapy, including IRI
  FOLFOX [62, 90] + Bmab [85, 91]
  CapeOX (note 2) [92] + Bmab [85]
  SOX + Bmab
  FOLFOX + Cmab/Bmab

(c) When the patient has become refractory or intolerant to the first-line therapy, including 5-FU, OX, and IRI
  IRI + Cmab/Pmab [93]
  Cmab/Pmab [94–97]
2. Patients not appropriate for intensive therapy
  BSC
  If possible, consider the optimal regimen

Third-line and subsequent therapies

- The following regimens should be considered for their-line and subsequent therapies
- IRI + Cmab/Pmab [93]
- Cmab/Pmab [94–97]
- Regorafenib [98]
- TAS-102 [99, 100].

Comments

① Careful attention is required when using IRI to treat patients with constitutional jaundice, such as that caused by Gilbert’s syndrome, or those with high serum bilirubin values. Associations between genetic polymorphisms of enzymes that metabolize IRI (UGT1A1) and toxicity have been suggested (see attached Side Memo 2).
② Although hepatic arterial infusion therapy shows high response rates for liver metastasis, it does not demonstrate any survival benefit compared with systemic chemotherapy [101]. (CQ-17)
③ The efficacy and safety of Rmab are evaluated based on a RAISE study*, which has been approved for use in combination with 5-FU, *l*-LV, and IRI in Japan. In addition, as described in the drug package insert, the efficacy and safety have not been established in postoperative adjuvant chemotherapy or the primary treatment. We should also consider that the RAISE study has been performed only among patients with PS 0 or 1, without evaluating the safety and efficacy in patients with PS 2–4.
④ The efficacy and safety of Regorafenib are evaluated on the basis of a CORRECT study [98]. As described in the drug package insert, the efficacy and safety have not been established in the primary or secondary treatments. We should also consider that the safety and efficacy have only been confirmed for patients with PS 0 or 1, but not for those with PS 2–4.
⑤ The efficacy and safety of TAS-102 are evaluated on the basis of J-003 study and RECOURSE study. As described in the drug package insert, the efficacy and safety have not been established in the primary or secondary treatments [99, 100]. We should also consider that the RECOURSE study has been performed only among patients with PS 0 or 1, without evaluating the safety and efficacy in patients with PS 2–4.
⑥ MSI-high (microsatellite-instability-high) can be observed in patients with colorectal cancer having Lynch syndrome caused by the mutations in the germlines of MMR (mismatch repair) genes or sporadic colorectal cancer caused by the acquired aberrant methylation of MLH1 genes. In general, it is found in approximately 5% of colorectal cancers [102]. Evidence for the effectiveness of chemotherapy only for MSI-high unresectable colorectal cancer has not been established, therefore under the current circumstances the common regimens for sporadic colorectal cancer are indicated for these patients. Some studies have recently reported that MSI-high may predict the poor prognosis of unresectable colorectal cancer, along with the effects of anti-PD-1 antibodies [103], however anti-PD-1 antibodies are not currently approved for MSI-high unresectable colorectal cancer in Japan. According to the NCCN guidelines as of February 2016, conducting the MMR gene test and MSI test are recommended, as the screening for Lynch syndrome in colorectal cancer patients under the age of 70 as well as patients over 70 met Bethesda guidelines, and as a good prognosis factor and a predictor of the ineffectiveness of postoperative adjuvant 5-FU monotherapy for Stage II colon cancer [104]. In Japan, the MSI test is approved only for patients suspected of having Lynch syndrome, the diagnostic procedures of Lynch syndrome described in “JSCCR Guidelines 2016 for the Clinical Practice of Hereditary Colorectal Cancer.” [105].

Note 1: FOLFOX—infusional 5-FU + LV + OX
Note 2: CapeOX—Cape + OX
Note 3: SOX—S-1 + OX
Note 4: FOLFIRI—infusional 5-FU + LV + IRI
Note 5: FOLFOXIRI—infusional 5-FU + LV + IRI + OX
Note 6: IRIS—S-1 + IRI

Chapter 6: Radiotherapy

- Radiotherapy is used to treat patients with locally advanced rectal cancer either as adjuvant therapy after surgery to prevent recurrence or before surgery to reduce tumor volume and preserve the anal sphincter, and also as palliative care to relieve the symptoms and prolong the survival time of patients with unresectable colorectal cancer who have symptomatic lesions.

1. Adjuvant radiotherapy
  Adjuvant radiotherapy is classified into three categories, according to the timing of surgery and radiation therapy: preoperative radiotherapy, intraoperative radiotherapy, and postoperative radiotherapy.
  The purpose of adjuvant radiotherapy is to improve the local control rate and the survival rate of rectal cancer patients. The purpose of preoperative radiotherapy includes improving the anal sphincter preservation rate and improving the resection rate. However, insufficient evidence of improved survival has been found to make this the objective of adjuvant radiotherapy.
  Preoperative radiotherapy is indicated for patients with T stage clinically diagnosed as “invasion depth cT3 (SS/A) or deeper or cN-positive”; postoperative radiotherapy is indicated for patients with T stage pathologically diagnosed after surgery as “invasion depth cT3 (SS/A) or deeper or pN-positive, where the existence of a surgical dissection plane positive (RM1) or penetration of the surgical dissection plane by the cancer (RMX) is unclear”; and intraoperative radiotherapy is indicated for “surgical dissection plane positive (RM1) or penetration of the surgical dissection plane by the cancer (RMX) is unclear”.
  Radiotherapy is delivered with a linear accelerator, where electron beams are used for intraoperative radiotherapy and photon beams for external radiotherapy.

Comments

① Preoperative radiotherapy (CQ-18)
  1. Preoperative radiotherapy has the following advantages: seeding during surgery can be prevented by inactivating lesions with irradiation; a high percentage of tumor cells are normo-oxic and radiosensitive, because blood flow to the tumor is maintained; there has been little damage to the digestive tract, since the small bowel is not fixed within the pelvic cavity, thereby resulting in low radiation-induced delayed toxicity, which means a less toxic postoperative setting; improvement in the R0 resection rate and anal sphincter preservation can be expected because of tumor size reduction [106].
  2. Preoperative radiotherapy has the following disadvantages: early-stage patients may be subjected to overtreatment and postoperative complications may increase.
  3. Twelve phase III clinical trials of preoperative radiotherapy (without chemotherapy) have been reported [106], and in five of the 12 randomized controlled trials the local control rate in the group that received preoperative radiotherapy was significantly higher than in the surgery alone group. However, an improvement in the survival rate was observed in only one trial [107].
  4. Two meta-analyses of radiotherapy showed improvement in the local control rate compared to surgery alone, and improvement in the survival rate in the groups that received doses of 30 Gy or more. However, there is controversy as to whether there is improvement in the survival rate [108, 109].
  5. Trials of short-course radiotherapy with 5 Gy per fraction have been conducted, mainly in Europe [107, 110]. Because the late effects of radiation depend on the fraction size, long-term follow-up for late adverse effects, such as anal dysfunction and bowel dysfunction, is necessary.
  6. In the Dutch CKVO 95-04 trial, which compared preoperative radiotherapy (25 Gy delivered in five fractions in one week) + TME and TME alone to investigate the significance of adding short-course radiotherapy to TME, the 5- and 10-year local control rates were significantly higher in the combination therapy group, but there was no significant difference between the two groups in the 5- and 10-year survival rates [110–112]. The incidences of sexual dysfunction and bowel dysfunction were higher in the preoperative radiation combination therapy group than in the surgery-alone group [113, 114].
  7. The effect of preoperative radiotherapy in reducing the size of the primary tumor may enable sphincter preservation. When the purpose of the preoperative radiotherapy is sphincter preservation, it is desirable to perform surgery after allowing an appropriate period for the tumor to decrease in size (6–8 weeks after the completion of radiotherapy) [115].
  8. In Europe, four randomized controlled trials, including the EORTC trial, were performed to investigate the usefulness of adding chemotherapy to preoperative radiotherapy. The incidence of acute-phase adverse events was significantly higher in the preoperative chemoradiotherapy groups, but the pathologic complete response rates (pCR) were significantly higher than in the preoperative radiotherapy alone groups. In two trials, the exception being the short-course radiotherapy trial, the local recurrence rate was significantly lower in the preoperative chemoradiotherapy group, and there was no significant difference between the two groups in terms of sphincter preservation or survival rate [111–118].
  9. In a randomized controlled trial that compared preoperative chemoradiotherapy and postoperative chemoradiotherapy, there was no significant difference in the 5-year survival rate, but the local recurrence rate and incidence of grade 3 or higher adverse events were significantly lower in the preoperative chemoradiotherapy group. Among the patients in whom abdominoperineal resection (APR) was considered necessary at the time of enrollment, the percentage of patients in whom sphincter preservation was possible was significantly higher in the preoperative chemoradiotherapy group [119].
  10. A randomized controlled trial of 5-FU versus Cape combination chemotherapy in the preoperative chemoradiotherapy indicated that the two drugs had the same level of efficacy and safety [120, 121]. NCCN Guidelines allow the use of either 5-FU or Cape as standard combination chemotherapy in the preoperative chemoradiotherapy. The indications and use of Cape as an adjuvant therapy for rectal cancer has been approved for use under health insurance in Japan as of August 2016.
  11. In randomized controlled trials into the efficacy of adding OX to pyrimidine fluoride as a combination chemotherapy in the preoperative chemoradiotherapy, OX increased harmful phenomena in three tests, but demonstrated no efficacy in regard to pCR ratio, localized control ratio and survival [120, 122–124]; moreover, in one test, although there was no difference in harmful phenomena and no analysis was done into disease-free survival at the primary endpoint, the pCR ratio was significantly higher [125].

2. Palliative radiotherapy
  - Intrapelvic lesions (CQ-19)
    The purpose of palliative radiotherapy for intrapelvic lesions is to relieve symptoms such as pain, hemorrhage, and bowel movement disorders caused by intrapelvic tumors.
    The target volume includes the tumor that is causing the symptoms.

[Dose and fractionation]

- A total dose of 45 Gy to 50 Gy is administered in 1.8–2.0 Gy fractions.
- Depending on the patient’s general condition, such as performance status, and the severity of the symptoms, radiotherapy may be completed in a shorter term with a larger fraction size, for example 30 Gy in 10 fractions over 2 weeks.

(b) Extrapelvic lesions
  1. Bone metastases
    The purpose of palliative radiotherapy for bone metastases is to achieve pain relief, prevent pathological fractures, and prevent and treat spinal cord paralysis.
    The target volume includes the metastatic bone lesions causing the symptoms.

[Dose and fractionation]

- Local field radiotherapy, such as 30 Gy in 10 fractions and 20 Gy in five fractions, is widely performed.

(2) Brain metastases
  See the section on hematogenous metastases (Chapter 4).

[Dose and fractionation]

- When whole brain radiotherapy is performed, 30 Gy in 10 fractions is the standard treatment. If long-term survival is expected, fractionated radiotherapy, such as 37.5 Gy in 15 fractions and 40 Gy in 20 fractions, is considered.
- When stereotactic radiosurgery is performed, a peripheral dose of 16 Gy to 25 Gy is delivered in a single fraction.

Chapter 7: Palliative care

- Palliative care is a general term for palliative treatment of various mental and physical symptoms related to cancer.
- Palliative care extends from the time the diagnosis of cancer is made to the end stage, and different care should be provided depending on the disease stage and symptoms.
- In principle, cancer treatment should be performed under conditions in which symptom relief is achieved [126], and palliative care should be started at the same time as surgical treatment and chemotherapy.
- Palliative care to improve the QOL of patients with end-stage colorectal cancer includes:

1. Pain relief
2. Surgical treatment
3. Chemotherapy
4. Radiotherapy
5. Counseling for psychiatric symptoms

Chapter 8: Surveillance after surgery for colorectal cancer

1. Surveillance for recurrence after curability A resection of colorectal cancer

1. Consideration should be given to periodic endoscopic examination for recurrence at the site of local resection or anastomosis in pStage 0 [pTis (M) cancer] cases. Surveillance for recurrence in other organs is not necessary.
2. pStage I–pStage III cases should be surveyed for recurrence in the liver, lungs, local area, anastomosis, lymph nodes, peritoneum, etc. The following points should be noted.
  In principle, the duration of surveillance is 5 years after surgery, but the surveillance examinations should be scheduled at shorter intervals during the first 3 years after surgery.
  It should be noted that there is a higher incidence of lung metastasis and local recurrence in rectal cancer than in colon cancer.
  As a general rule, the duration of surveillance for anastomotic recurrence is until 3 years after surgery.
  The following is an example of a surveillance schedule after curative resection of Stage I to Stage III colorectal cancer that was designed on the basis of the results of a retrospective investigation of such factors as the common sites and incidence of recurrence and the efficacy of treatment and the clinical practice in Japan (Fig. 7).

**Fig. 7:**
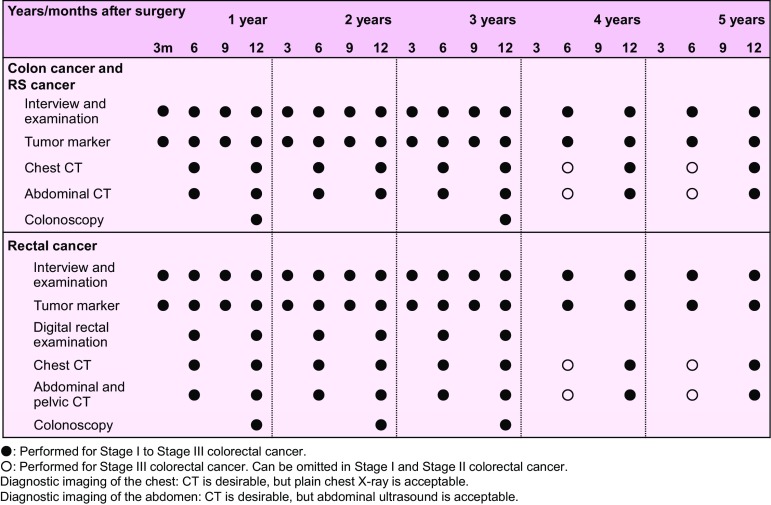
An example of a surveillance schedule after curative resection of pStage I to pStage III colorectal cancer
3. Surveillance after curability B resection of colorectal cancer and after resection of recurrent tumors.
  1. The same surveillance method as for Stage III colorectal cancer is used. It should be noted that recurrence and re-recurrence are common in organs previously operated on.
  2. In cases allocated curability B due to R1 resection, close surveillance schedule should be planned for organs in which residual cancer is suspected.
4. Surveillance of metachronous multiple cancer
  Colonoscopy is performed for surveillance of metachronous multicentric colorectal cancer.

Comments

① Aim of surveillance
  The aim of surveillance is to improve the patient’s prognosis by early detection and treatment of recurrences. Meta-analyses of RCTs conducted in Europe and the United States have shown that surveillance after curative surgical resection of colorectal cancer contributes to improving the resection rate of recurrent tumors and to improving the prognosis [127–131] (CQ-20-1).
② Recurrence rate, sites of recurrence, times of recurrence
  The results of the project study by the JSCCR are shown in Figs. 8, 9 and Tables 10, 11, 12, and 13. The subjects were patients who underwent curative resection of colorectal cancer between 1991 and 1996 at the 14 institutions that participated in the project, and the follow-up period was 6–11 years.

**Fig. 8:**
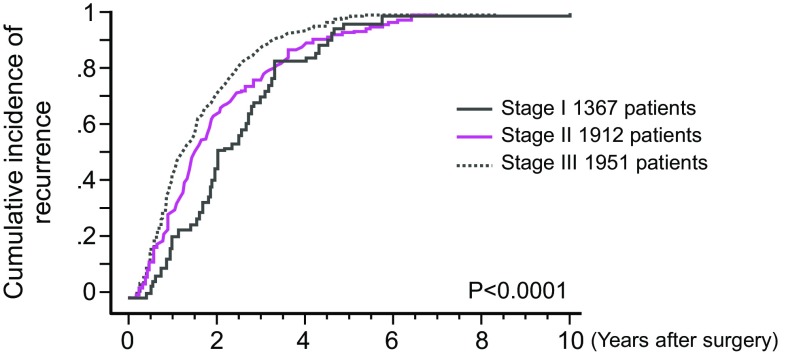
Graph of the cumulative incidence of recurrence according to stage (project study by the JSCCR: patients in years 1991–1996)

**Fig. 9:**
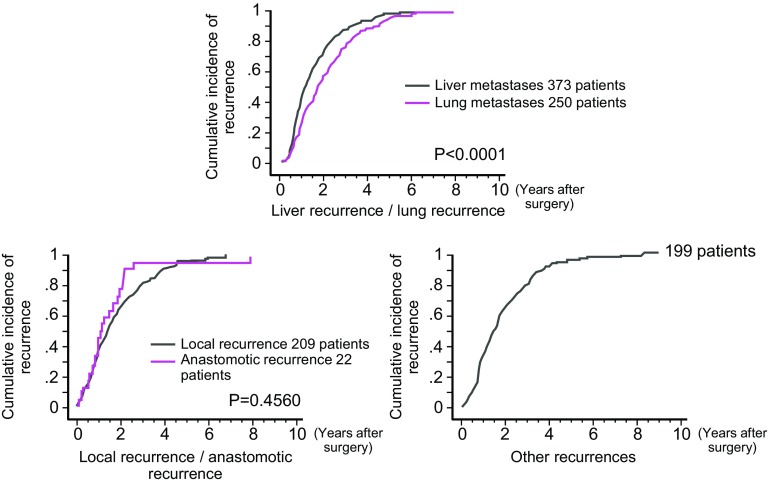
Graph of the cumulative incidence of recurrence according to the site of recurrence (project study by the JSCCR: patients in years 1991–1996)

**Table 10:** Recurrence rate after curative resection of colorectal cancer according to stage and cumulative incidence of recurrence according to the number of years after surgery

Stage (no. of patients)	Recurrence rate (no. of patients with recurrence)	Cumulative incidence of recurrence according to the no. of years after surgery (cumulative no. of patients with recurrence)			Percentage of patients experiencing recurrence more than 5 years after surgery among all patients (no. of patients)
		3 years	4 years	5 years	
I	3.7%	68.6%	82.4%	96.1%	0.15%
(1367)	(51)	(35)	(42)	(49)	(2)
II	13.3%	76.9%	88.2%	92.9%	0.94%
(1912)	(255)	(196)	(225)	(237)	(18)
III	30.8%	87.0%	93.8%	97.8%	0.67%
(1957)	(600)	(522)	(563)	(587)	(13)
All	17.3%	83.2%	91.6%	96.4%	0.63%
(5230)	(906)	(753)	(830)	(873)	(33)

Project study of the JSCCR: patients in years 1991–1996

**Table 11:** Recurrence rate of Stage I colorectal cancer (RS cancer was counted as colon cancer)

Stage I	No. of patients	No. of patients with recurrence	Recurrence rate (%)	*p* value
Tumor location				
Colon	891	24	2.7	0.0056
Rectum	476	27	5.7	
Depth of tumor invasion				
SM	714	9	1.3	<0.0001
MP	653	42	6.4	
Tumor location and depth of tumor invasion				
Colon				
SM	528	7	1.3	0.0024
MP	363	17	4.7	
Rectum				
SM	186	2	1.1	0.0005
MP	290	25	8.6	

Project study of the JSCCR: patients in years 1991–1996

**Table 12:** Recurrence rate according to the site of the first recurrence after curative resection of colorectal cancer and cumulative incidence of recurrence according to the number of years after surgery

Site of first recurrence	Recurrence rate (no. of patients with recurrence (including overlaps)	Cumulative incidence of recurrence according to the number of years after surgery (cumulative no. of patients with recurrence)			Percentage of patients experiencing recurrence more than 5 years after surgery among all patients (no. of patients)
		3 years	4 years	5 years	
Liver	7.1% (373)	87.9% (328)	94.1% (351)	98.7% (368)	0.10% (5)
Lung	4.8% (250)	78.0% (195)	88.8% (222)	94.8% (237)	0.25% (13)
Local	4.0% (209)	80.9% (169)	90.4% (189)	96.2% (201)	0.15% (8)
Anastomotic	0.4% (22)	95.5% (21)	95.5% (21)	95.5% (21)	0.02% (1)
Other	3.8% (199)	79.4% (158)	91.0% (181)	95.5% (190)	0.17% (9)
All (5230)	17.3% (906)				

Project study of the JSCCR: patients in years 1991–1996

**Table 13:** Comparison between the recurrence rates of colon cancer and rectal cancer according to the site of the first recurrence (RS cancer was counted as colon cancer)

Site of recurrence	Colon cancer (3583 patients)	Rectal cancer (1647 patients)	*p* value
Liver	7.0% (252)	7.3% (121)	NS
Lung	3.5% (126)	7.5% (124)	<0.0001
Local	1.8% (64)	8.8% (145)	0.0001
Anastomotic	0.3% (9)	0.8% (13)	0.0052
Other	3.6% (130)	4.2% (69)	NS
All	14.1% (506)	24.3% (400)	<0.0001

Project study of the JSCCR: patients in years 1991–1996
(1) Times of the recurrences and sites of the recurrences (Fig. 9, Tables 10, 12, 13).
  More than 80% of the recurrences were detected within 3 years after surgery, and more than 95% of the recurrences were detected within 5 years after surgery.
  The overall incidence of recurrence more than 5 years after surgery was less than 1%.
  Among lung recurrences, 5% of recurrences were detected more than 5 years after surgery.
  More than 95% of the anastomotic recurrences were detected within 3 years after surgery.
  Local recurrence and lung recurrence were more frequent in rectal cancer than in colon cancer.
  There have been reports regarding recurrences after curative resection in Europe and the United States showing that approximately 50% of the recurrences were detected within 1 year after surgery, that approximately 70% of the recurrences were detected within 2 years after surgery [132, 133]; and that in most patients the recurrences were detected within 5 years after surgery [133].
(2) Characteristics of recurrence according to pStage (Fig. 8, Tables 10, 11)
1. pStage I
  The recurrence rate of pT1 (SM) cancer was approximately 1% in both colon cancer and rectal cancer.
  The overall recurrence rate of pT2 (MP) cancer was 6.4%, and it was 5.0% in colon cancer and 8.3% in rectal cancer.
  Two-thirds of the recurrences were detected within 3 years after surgery, and the overall incidence of recurrence more than 5 years after surgery was less than 0.2% among all patients.
2. pStage II, pStage IIIa, and pStage IIIb
  The recurrence rate increased with the Stage.
  78–90% of recurrences were detected within 3 years after surgery, and the overall incidence of recurrence more than 5 years after surgery was less than 1% among all patients.
③ Surveillance of metachronous multiple primary cancer
  A past history of colorectal cancer, regardless of stage, is a risk factor for metachronous colorectal cancer [134].
  The recommended interval between colonoscopy ranged from 1 to 5 years, depending on the report [135].
  The need for surveillance targeting multiple cancers should be determined by distinguishing hereditary colorectal cancer [105]. There is little evidence of a need for periodic minute examinations for cancer in other organs following surgery for sporadic colorectal cancer (CQ-20-2).

Clinical questions

CQ-1: What are the indication criteria for additional treatment after endoscopic resection of pT1 (SM) [26]? (Fig. 10)

① Surgical resection is preferable when the vertical margin is positive (recommendation/evidence level 1C).
② If any of the following findings is observed during histological examination of the resected specimen, intestinal resection with lymph node dissection is considered as an additional treatment (evidence level B).
  1. Depth of SM invasion ≥1000 µm
  2. Vascular invasion positive
  3. Poorly differentiated adenocarcinoma, signet-ring cell carcinoma, or mucinous carcinoma [136]
  4. Grade 2/3 budding at the site of deepest invasion [136]

**Fig. 10:**
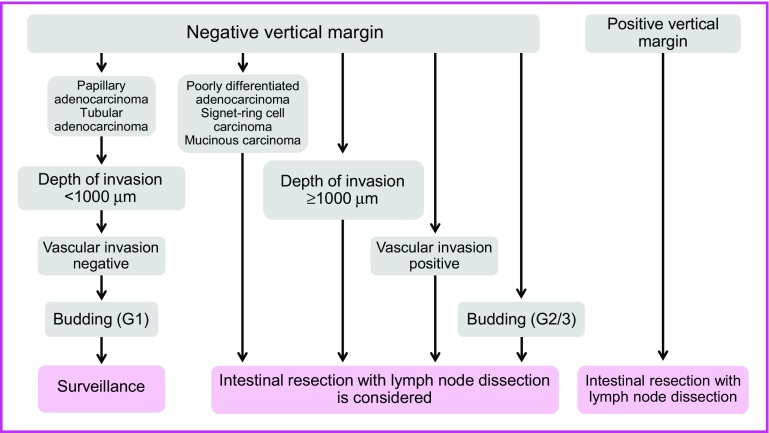
Treatment strategies for pT1 (SM) cancer after endoscopic resection

Note

- “Vertical margin-positive” means that carcinoma is exposed at the submucosal margin of the resected specimen.
- Depth of SM invasion is measured by the method described in Side Memo 1 (Fig. 11).

**Fig. 11:**
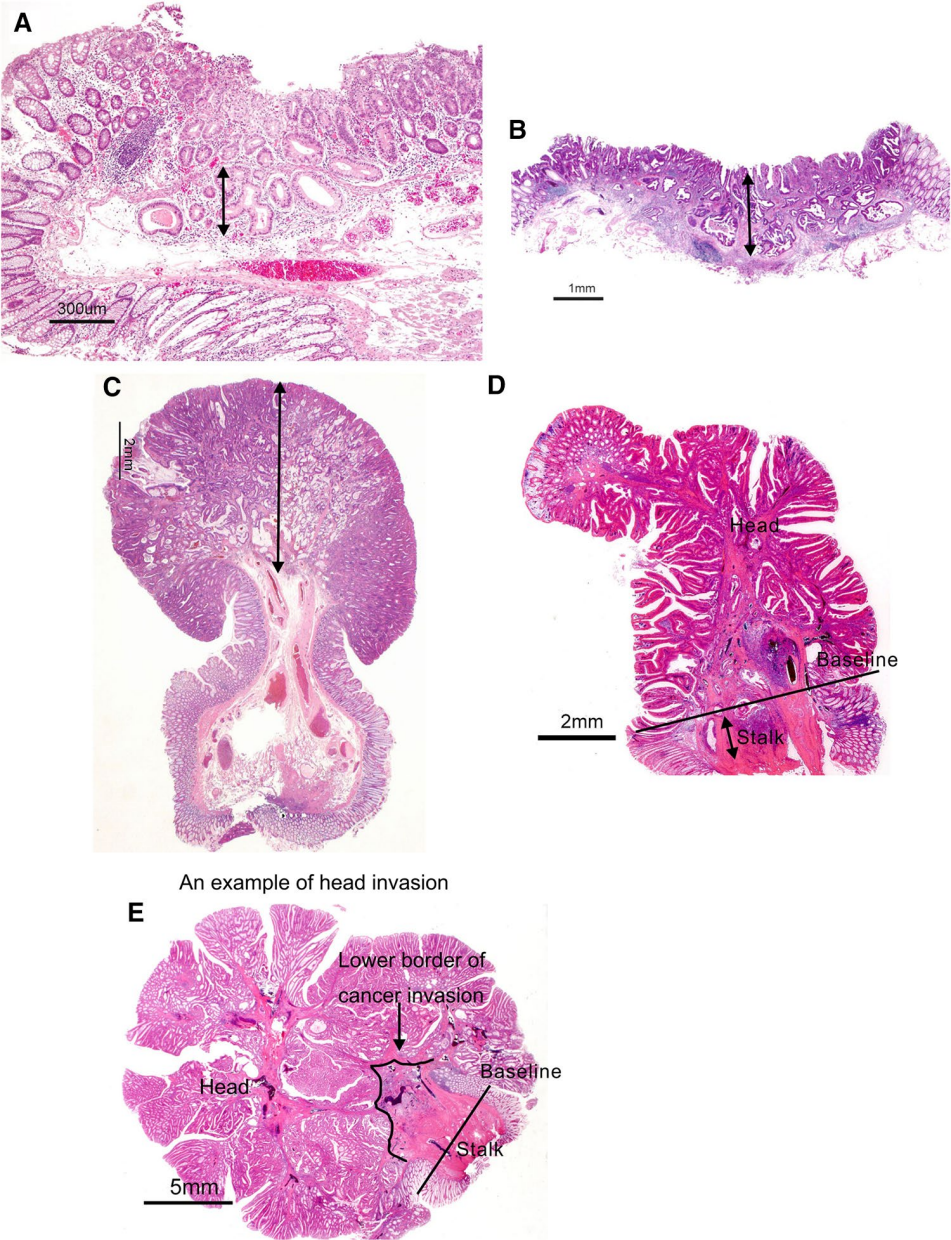
Method for measuring depth of SM invasion. **a** When it is possible to identify or estimate the location of the muscularis mucosae, depth of SM invasion is measured from the lower border of the muscularis mucosae. **b**, **c** When it is not possible to identify or estimate the location of the muscularis mucosae, depth of SM invasion is measured from the surface layer of the muscularis mucosae. Sessile lesion (**b**), Pedunculated lesion (**c**). **d** For pedunculated lesions with tangled a muscularis mucosae, depth of SM invasion is measured as the distance between the point of deepest invasion and the reference line, which is defined as the boundary between the tumor head and the stalk. **e** Invasion by pedunculated lesions that is limited to within the head is defined as “head invasion”
- Vascular invasion consists of lymphatic and venous invasion (Figs. 12, 13, 14).

**Fig. 12:**
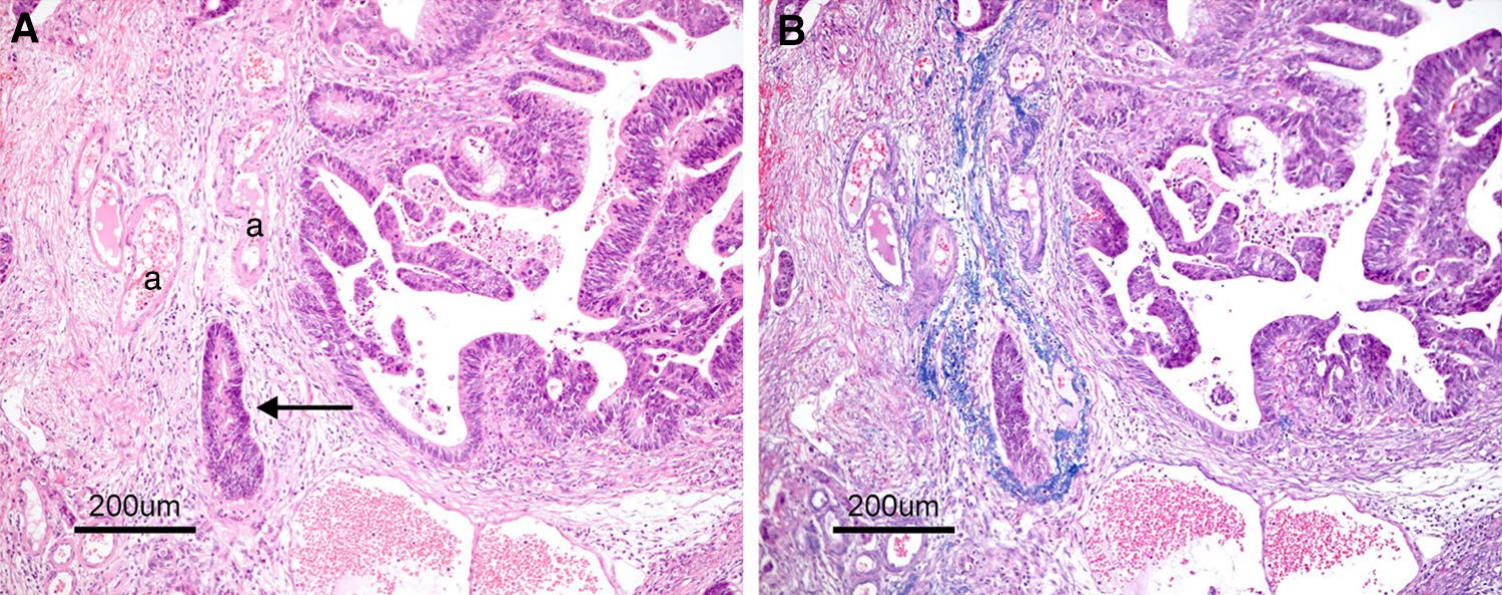
Venous invasion (*arrow* in **A**). **A** Located in the vicinity of an artery (*a*). **B** Elastic fibers in the vein wall have become clear by Victoria blue staining

**Fig. 13:**
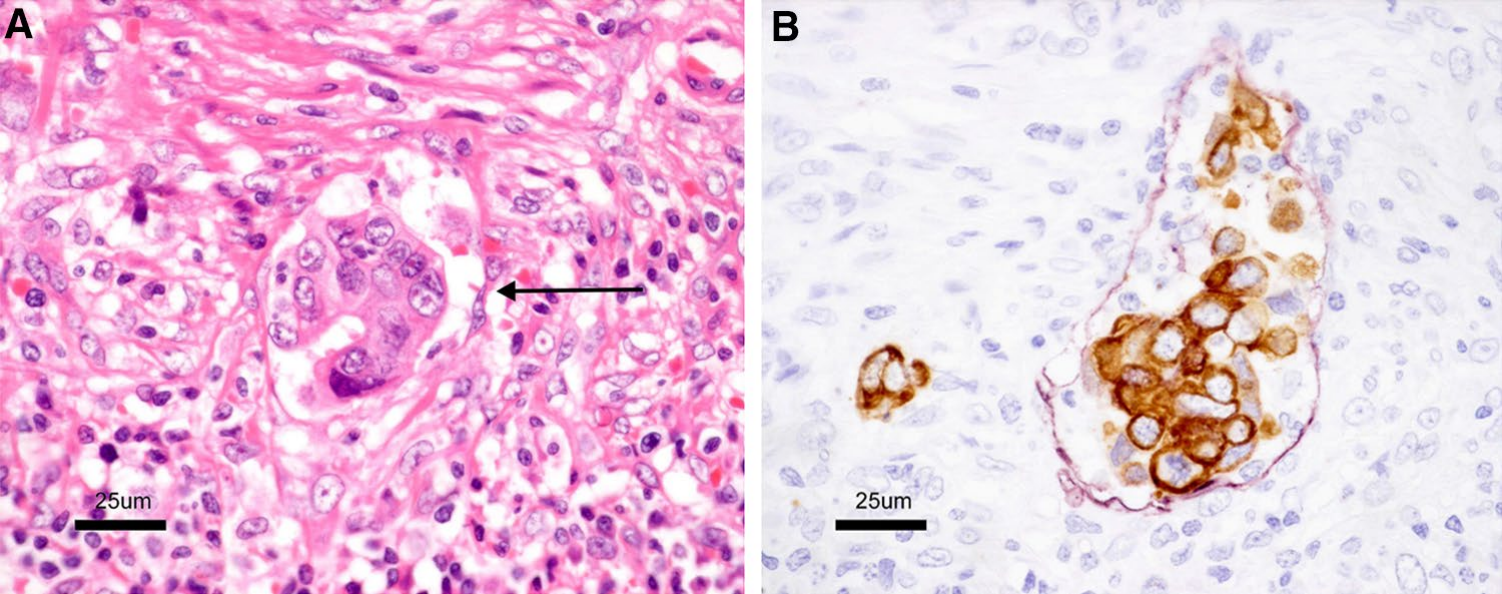
Lymphatic invasion (*arrow* in **a**). **a** A cancer cell nest is visible in the interstitial space. **b** Double staining for cytokeratin and D2-40. Cancer cells are stained *brown*, and the lymphatic endothelium is stained *purplish red*

**Fig. 14:**
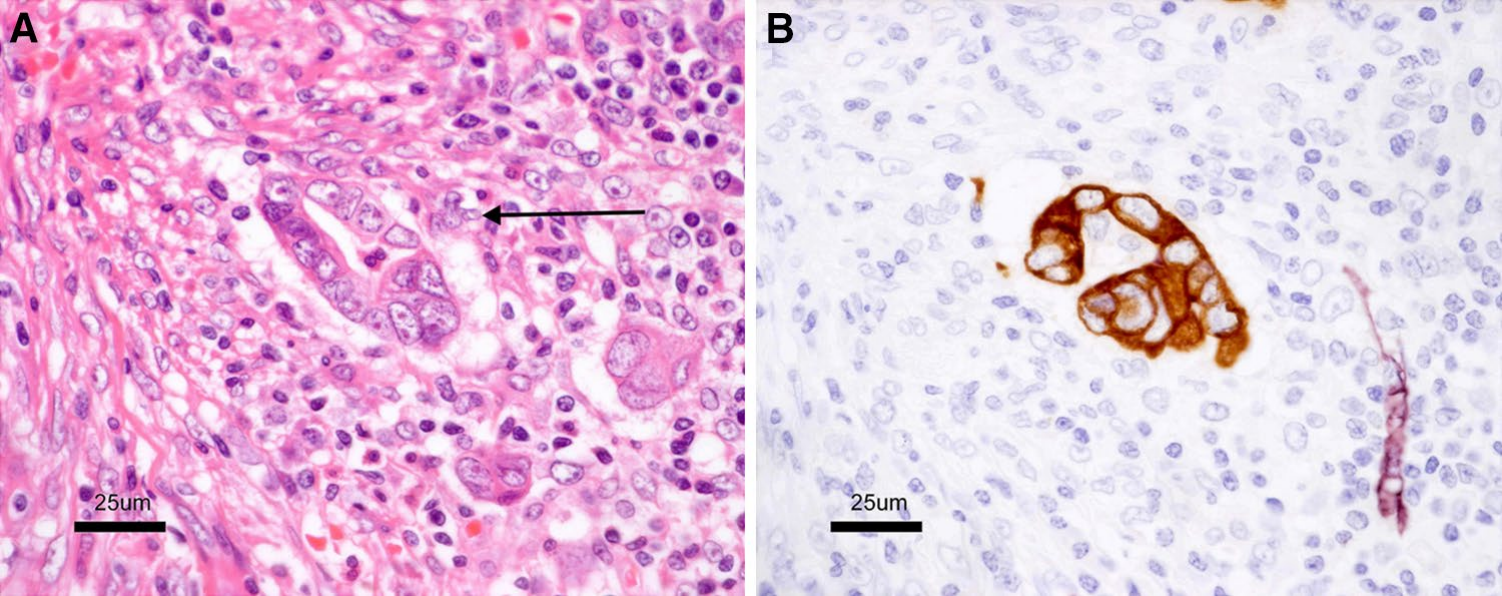
Space formed by artifacts during preparation of the specimen (*arrow* in **a**). **a** A cancer cell nest is visible in the interstitial space. **b** Double staining for cytokeratin and D2-40. The interstitial space is D2-40-negative
- The method of assessing budding is described in Fig. 15.

**Fig. 15:**
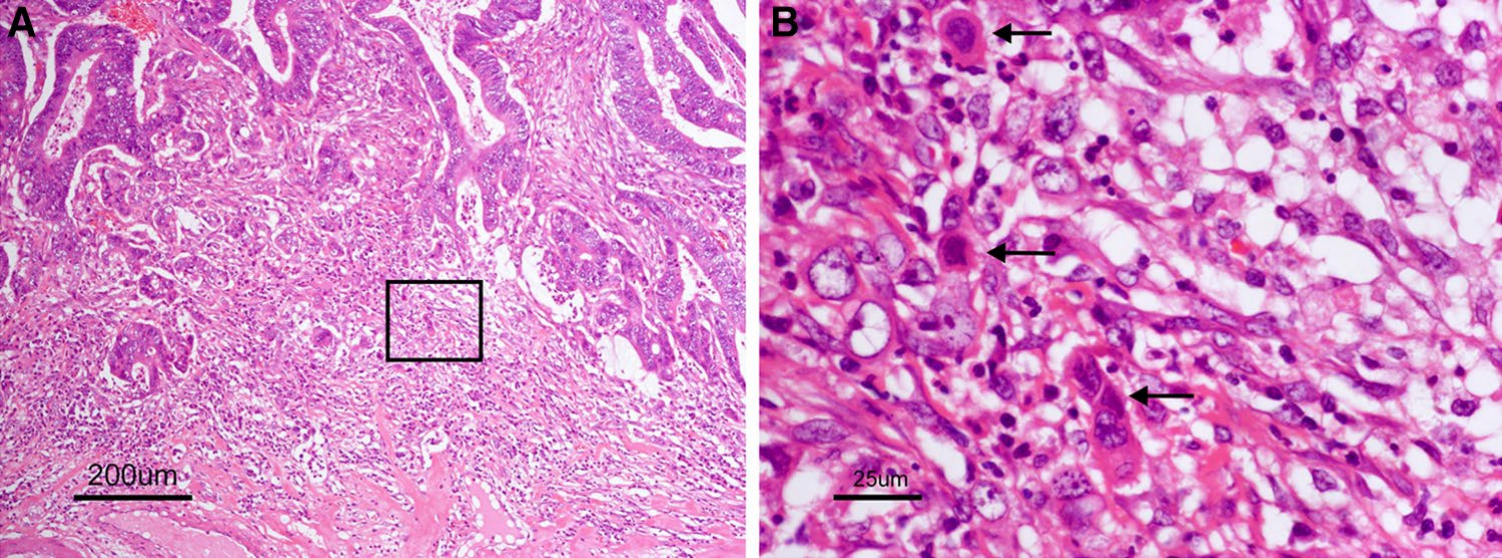
Budding (*arrow* in **b**). A cancer cell nest consisting of one or fewer than five cells that has infiltrated the interstitium at the invasive margin of the cancer is seen. **b** is the square area in **a**

The principle for treatment of pT1 (SM) carcinomas, which are invasive carcinomas, is intestinal resection with lymph node dissection. However, some pT1 (SM) carcinomas have a very low risk of metastasis, and the purpose of these criteria is to minimize the need for additional resections that eventually result in overtreatment of such patients. While no diagnostic methods make it possible to predict lymph node metastasis (pN) without fail, the degree of risk of metastasis can be used as a basis for determining whether or not to perform additional treatment.

Factors such as the depth of submucosal invasion (SM invasion depth) [137], histological type, such as poorly differentiated adenocarcinoma, signet-ring cell carcinoma, and mucinous carcinoma [136], the presence of a poorly-differentiated area and muconodules at the site of deepest invasion, budding, and vascular invasion, have been reported to be risk factors for regional lymph node metastasis by pT1 (SM) carcinoma [136, 137].

The above criteria for determining whether additional treatment is indicated were prepared based on the following three criteria for performing additional intestinal resection of pT1 (SM) carcinoma described in the “Japanese Classification of Colorectal Carcinoma” (2nd edition, 1980): [(1) Obvious intravascular carcinoma invasion; (2) Poorly differentiated adenocarcinoma or undifferentiated carcinoma; (3) Massive carcinoma invasion extending to the vicinity of the margin] [138]. The description of “Massive carcinoma invasion” in the 4th edition of the “Japanese Classification of Colorectal Carcinoma” was revised to the following more specific description in the 5th edition (1994): “Invasion deeper than ‘very shallow invasion’ (e.g., invasion exceeding approximately 200–300 μm)” [139].

Subsequent case series studies in Japan have shown that “200–300 μm” can be extended to 1000 μm [140]. According to the results of the project study by the JSCCR, the lymph node metastasis rate of colorectal carcinoma with an SM invasion depth of 1000 μm or more was 12.5% (Table 14) [137, 140]. However, not all cases with submucosal invasion deeper than 1000 μm necessarily require additional surgery. Approximately 90% of patients with a depth of invasion of 1000 μm or more did not have lymph node metastasis, and it is important to determine whether additional treatment is indicated after sufficiently considering other factors in addition to depth of SM invasion, such as whether other risk factors for lymph node metastasis are present, the physical and social background of the patient, and the patient’s wishes. As consensus has not yet been achieved within the Guideline Committee, indicators of strength of recommendation in the treatment criteria provided above have not been disclosed. Because budding was demonstrated to be an important risk factor for lymph node metastases in the project study by the JSCCR, additional intestinal resection has been added to the list of factors that should be considered in the previous edition. Furthermore, project research is currently underway into other histopathological factors. Multi-center joint research projects have produced reports providing the results of consideration into the appropriateness of these criteria [141–143]. None of the guidelines in other countries include depth of invasion or budding as criteria for additional treatment.

**Table 14:** Depth of invasion of sm cancer and lymph node metastasis (modified from Reference 201)

sm invasion distance (μm)	Pedunculated		Non-pedunculated	
	Number of lesions	*n* (+) (%)	Number of lesions	*n* (+) (%)
head invasion	53	3 (5.7)		
0 < *X*<500	10	0 (0)	65	0 (0)
500 ≤ *X* < 1000	7	0 (0)	58	0 (0)
1000 ≤ *X* < 1500	11	1 (9.1)	52	6 (11.5)
1500 ≤ *X* < 2000	7	1 (14.3)	82	10 (12.2)
2000 ≤ *X* < 2500	10	1 (10.0)	84	13 (15.5)
2500 ≤ *X* < 3000	4	0 (0)	71	8 (11.3)
3000 ≤ *X* < 3500	9	2 (22.2)	72	5 (6.9)
3500 ≤ *X*	30	2 (6.7)	240	35 (14.6)

The lymph node metastasis rate of patients with a depth of invasion of 1000 μm or above was 12.5%

All three lymph node metastasis-positive patients with head invasion were ly positive

CQ-2: What are the criteria for selecting endoscopic resection in regard to lesions with a maximum diameter of 2 cm or greater?

- Accurate preoperative endoscopic diagnosis is essential in endoscopic resection in regard to lesions with a maximum diameter of 2 cm or greater, and whether resection by EMR, piecemeal EMR, or ESD is indicated is determined after taking the operator’s skill in performing endoscopic resection into consideration. (Recommendation/Evidence level 1B)

Side Memo 1

- Method for measuring depth of SM invasion (Fig. 11)
  When it is possible to identify or estimate the location of the muscularis mucosae, depth of SM invasion is measured from the lower border of the muscularis mucosae of the lesion, regardless of the macroscopic type.
- When it is not possible to identify or estimate the location of the muscularis mucosae, the depth of SM invasion is measured from the surface of the lesion. The phrase “possible to identify or to estimate” means that there is no “deformity”, i.e., disarray, dissection, rupture, fragmentation, etc., of the muscularis mucosae as a result of SM invasion. If a deformed muscularis mucosa is used as the base line of the measurement, the depth of SM invasion may be underestimated. Although judging whether there is a “deformity” is not always straightforward, if a desmoplastic reaction is present around the muscularis mucosae, it is assumed to be “deformed.”
- For pedunculated lesions with a tangled muscularis mucosae, depth of SM invasion is measured as the distance between the point of deepest invasion and the reference line, which is defined as the boundary between the tumor head and the stalk (the boundary between the tumor area and the non-tumor area in the mucosa). Invasion by pedunculated lesions that is limited to within the head is defined as “head invasion.”
- Method for assessing vascular invasion (Figs. 12, 13, 14)
- Attention to arteries is a key factor in assessing venous invasion. Venous invasion is highly likely when a circular, semicircular, or oblong cancer cell nest with regular margins is located in the vicinity of an artery and distant from the main lesion. If such a cancer cell nest is surrounded by venous wall structures (such as internal elastic membrane or perivascular smooth muscle), it can be concluded to represent venous invasion. However, the venous wall structures are often displaced or obliterated by the cancer cell nest, and it is difficult to recognize in hematoxylin and eosin stained sections.
- The presence of cancer cells and cancer cell nests in the interstitial space suggests lymphatic invasion. A space filled with lymph and lymphocytes is especially likely to be a lymph vessel. When endothelial cells are identified around the space, the space can be concluded to represent a lymph vessel. However, it is often difficult to identify endothelial cells in specimens stained with hematoxylin and eosin, and spaces may be artifacts created during the process of preparing the specimen.
- As stated above, evaluation of vascular invasion, which is an important indicator for determining treatment strategies for SM cancer, is often difficult in hematoxylin and eosin stained specimens. Special staining methods are useful for evaluating vascular invasion, such as elastica van Gieson staining or Victoria blue staining for venous invasion, and D2-40 immunostaining for lymphatic invasion.
- Method for the assessing tumor budding (Fig. 15)

[Definition of tumor budding] [136] A cancer cell nest consisting of one or fewer than five cells that infiltrates the interstitium at the invasive margin of the cancer.

[Grade of budding] After selecting one field where budding is the most intensive, number of buddings is counted in a field measuring 0.785 mm^2^ observed through a 20× objective lens (WHK 10× ocular lens). Depending on the number of buddings, Grade of budding is defined as follows:

Grade 1: 0–4
Grade 2: 5–9
Grade 3: 10 or more

- The lymph node metastasis rate by Grade 2/3 tumors is significantly higher than by Grade 1 tumors. A multi-center study conducted by the Budding Investigation Project Committee (2005–) of the JSCCR in which Grade 1 was defined as “low grade” and Grade 2/3 as “high grade” showed that “high grade” is an independent predictor of lymph node metastasis.

CQ-3: what cautions should be noted when using colorectal ESD to implement endoscopic resection of colonic lesions?

- While ESD is used in cases of “early-stage malignant tumors,” accurate preoperative endoscopic diagnosis and the level of skill of the operator in regard to endoscopic resection should be considered before deciding to proceed. (Recommendation/Evidence level 1B)

CQ-4: is laparoscopic surgery for colorectal cancer effective?

- According to randomized controlled trials held overseas and the Cochrane Database of Systematic Reviews, the safety and long-term outcome of laparoscopic surgery in cases of colonic and RS cancers are similar to those in open surgery. As D3 dissection is difficult under laparoscopic conditions, laparoscopic surgery for cStage II–cStage III disease should be implemented when it is considered that the individual surgical team is sufficiently experienced. Laparoscopic surgery is also difficult in patients with transverse colon cancer, in severely obese patients, and in patients with severe adhesions.
- The efficacy and safety of laparoscopic surgery for rectal cancer has not been established. Ideally, appropriately planned clinical trials should be implemented. (Recommendation/Evidence level 1B).

CQ-5: resection of the primary tumor in patients with unresectable distant metastases

- The efficacy of primary tumor resection in cases with unresectable distant metastasis differs depending on individual factors such as symptoms caused by the primary lesion, the state of distant metastasis, the patient’s general condition, etc.

① If symptoms exist as a result of the primary tumor, which are difficult to control using other therapies, and the resection is not significantly invasive, primary tumor resection and early systemic chemotherapy is recommended. (Recommendation/Evidence level 1C)
② For cases in which no symptoms are caused by the primary tumor; however, the efficacy of resecting the primary tumor has not been established.

CQ-6: in cases where peritoneal dissemination is noted, is it effective to resect peritoneal dissemination at the same time as the primary lesion?

- The efficacy of resecting peritoneal dissemination has not been proven. Some cases of long-term survival have been reported in which localized dissemination (P1, P2) was resected alongside the primary tumor, suggesting that if the resection is not significantly invasive, then the peritoneal dissemination should be resected at the same time as the primary tumor (recommendation/evidence level 2D).

CQ-7: what are the indications for resection for cases in which metastasis is simultaneously noted in the liver and the lungs?

- The efficacy of resection in patients who have liver and lung metastases at the same time has been shown, and thus resection should be considered for patients with resectable liver and lung metastases.

However, there are insufficient data to determine the indication criteria for surgery. It is necessary to obtain informed consent after informing the patient of the rather low cure rate and the absence of outcome predictors (recommendation/evidence level 2D).

CQ-8: is adjuvant chemotherapy effective subsequent to distant metastatic lesion resection?

- The efficacy and safety of adjuvant chemotherapy subsequent to distant metastatic lesion resection in cases of colorectal cancer have not yet been established. Ideally, appropriately planned clinical trials should be implemented. (Evidence level C)

CQ-9: is resection of liver/lung metastasis effective, if it becomes possible as a result of the effects of chemotherapy?

- Resection should be performed for cases in which chemotherapy has successfully made localized metastasis to the liver or lungs operable (recommendation/evidence level 2D).

CQ-10: what are the surgical indications in cases of local recurrence of rectal cancer?

- Resection should be considered for local recurrence of rectal cancer when R0 resection is considered possible (recommendation/evidence level 2D).

CQ-11: is preoperative adjuvant chemotherapy effective in cases of operable liver metastasis?

- The efficacy and safety of preoperative chemotherapy for resectable liver metastases has not been established. It should be evaluated in properly designed clinical trials (evidence level D).

CQ-12: is heat coagulation therapy effective in regard to liver metastatic lesions?

① There are few reports indicating the efficacy of heat coagulation therapy, and as such, it is not recommended as a first choice of treatment (recommendation/evidence level 1C).
② Since heat coagulation therapy is accompanied by a high risk of local recurrence in cases of liver metastasis, resection should be initially considered wherever possible.

CQ-13: is postoperative adjuvant chemotherapy effective in patients aged 70 or over?

- Even in patients 70 years old or older, postoperative adjuvant chemotherapy is recommended if their PS is good, if the function of major organs is adequate, and if there are no complications that may be a risk for performing chemotherapy (recommendation/evidence level 1A).

CQ-14: should postoperative adjuvant chemotherapy for Stage II [26] colorectal cancer be carried out?

- The usefulness of postoperative adjuvant chemotherapy for Stage II colorectal cancer has not been proven, and it is recommended not to routinely administer adjuvant chemotherapy to all patients with Stage II colorectal cancer (recommendation/evidence level 1A).

CQ-15: is the appropriate duration of postoperative adjuvant chemotherapy six months?

- Although no definitive conclusion regarding the duration of postoperative adjuvant chemotherapy has been reached, the current standard duration of treatment by 5-FU-based adjuvant chemotherapy is 6 months (recommendation/evidence level 1A).

CQ-16-1: is concomitant therapy with molecular target drugs recommended as the primary treatment?

As long as there are no contraindications, usage in combination with bevacizumab, cetuximab or panitumumab is recommended (recommendation level/evidence level 1A).

CQ-16-2: is concomitant therapy with molecular target drugs recommended as the secondary treatment?

As long as there are no contraindications, consider a combination of the following molecular target drugs as the secondary treatment.

Bmab: Proposed regardless of the primary treatment regimen (2B),

Anti-EGFR antibody drugs: Not recommended for patients treated with anti-EGFR antibody in the primary treatment (1D),

Proposed for the patients treated without anti-EGFR antibody in the primary treatment (2C).

CQ16-3: is of regorafenib or TAS-102 therapy recommended for 3rd or later line treatment?

Propose both drugs for patients with PS0-1 who are failed or intolerant to the standard therapy. (1A)

Side Memo 2

- IRI and UGT1A1 genetic polymorphism

SN-38 is an active metabolite of IRI and the UGT1A1 gene encodes an intrahepatic metabolizing enzyme, which converts the active form SN-38 to the inactive form SN-38 G. In patients who are double heterozygotes for *6 and *28 or homozygotes for *6 or *28 of the UGT1A1 gene, the glucuronic acid conjugation capacity of UGT1A1 is known to be decreased and the metabolism of SN-38 to be delayed, and serious adverse drug reactions such as neutropenia may occur as a result. It is especially desirable to test for a UGT1A1 genetic polymorphism before administering IRI to patients with a high serum bilirubin level, elderly patients, patients whose general condition is poor (e.g., PS2), and patients in whom severe toxicity (especially neutropenia) developed after the last administration of IRI. On the other hand, because IRI toxicity cannot be predicted with certainty on the basis of the presence of a UGT1A1 genetic polymorphism alone, it is essential to monitor patients’ general condition during treatment and manage adverse drug reactions carefully regardless of whether a genetic polymorphism is detected.

CQ-17: is hepatic arterial infusion therapy effective in cases of liver metastases?

- Comparisons between hepatic arterial infusion therapy using fluoropyrimidine alone and systemic chemotherapy showed no clear difference in survival. The effectiveness of hepatic arterial infusion therapy in comparison with systemic chemotherapy using multi-drug combination has not been established (recommendation/evidence level 1C).

CQ-18: is preoperative chemoradiotherapy effective in patients with rectal cancer?

- In the USA and Europe, although preoperative chemoradiotherapy has lowered local recurrence rates in comparison with TME-only, reports suggest that it has not contributed to improved survival rates. In Japan, where surgical methods differ from the USA and Europe, the efficacy of preoperative chemoradiotherapy has not been established in regard to rectal cancers in which the lower margin of the tumor is closer to the anus than the peritoneal reflection (evidence level B).

CQ-19: is chemoradiotherapy effective for unresectable locally advanced and locally recurrent rectal cancer?

① In cases of locally advanced and locally recurrent rectal cancer determined likely to become R0 resectable as a result of tumor shrinkage following treatment, it is recommended that chemoradiotherapy, with the aim of resection, be used as opposed to radiotherapy alone (recommendation/evidence level 1B).
② Chemoradiotherapy should also be taken into consideration where the objective is relief of symptoms (recommendation/evidence level 1C).

CQ-20-1: is surveillance subsequent to curative surgery for colorectal cancer effective?

- It has been suggested that the efficacy of surveillance is its contribution to improving prognosis by allowing the early detection of recurrence, and as such, regular postoperative surveillance is desirable (recommendation/evidence level 1A).
- However, an optimal surveillance protocol incorporating the health economical point of view has not been sufficiently established.

CQ-20-2: is the surveillance of multiple cancers (multiple colorectal cancer or other organ cancer) effective subsequent to curative surgery for colorectal cancer?

① Metachronous colorectal cancer occurs more frequently in cases of colorectal cancer resection than in the general population, and as such, regular laparoscopic examination of the colon is recommended (recommendation/evidence level 1B).
② There is no indication that post-surgical surveillance targeting multiple cancers is effective. The appropriate course of action is to educate the patient regarding the need for regular cancer examinations and recommend periodic checkups (recommendation/evidence level 2C).
